# Transcriptomic analysis of differentially expressed genes in an *orange-pericarp* mutant and wild type in pummelo (*Citrus grandis*)

**DOI:** 10.1186/s12870-015-0435-3

**Published:** 2015-02-12

**Authors:** Fei Guo, Huiwen Yu, Qiang Xu, Xiuxin Deng

**Affiliations:** Key Laboratory of Horticultural Plant Biology (Ministry of Education), Huazhong Agricultural University, Wuhan, 430070 China

**Keywords:** Citrus, RNA-seq, Transcriptome profile, Carotenoid, qRT-PCR

## Abstract

**Background:**

The external colour of fruit is a crucial quality feature, and the external coloration of most citrus fruits is due to the accumulation of carotenoids. The molecular regulation of carotenoid biosynthesis and accumulation in pericarp is limited due to the lack of mutant. In this work, an *orange-pericarp* mutant (MT) which showed altered pigmentation in the pericarp was used to identify genes potentially related to the regulation of carotenoid accumulation in the pericarp.

**Results:**

High Performance Liquid Chromatography (HPLC) analysis revealed that the pericarp from MT fruits had a 10.5-fold increase of β-carotene content over that of the Wild Type (WT). Quantitative real-time PCR (qRT-PCR) analysis showed that the expression of all downstream carotenogenic genes was lower in MT than in WT, suggesting that down-regulation is critical for the β-carotene increase in the MT pericarp. RNA-seq analysis of the transcriptome revealed extensive changes in the MT gene expression level, with 168 genes down-regulated and 135 genes up-regulated. Gene ontology (GO) and KEGG pathway analyses indicated seven reliable metabolic pathways are altered in the mutant, including carbon metabolism, starch and sucrose metabolism and biosynthesis of amino acids. The transcription factors and genes corresponding to effected metabolic pathways may involved in the carotenoid regulation was confirmed by the qRT-PCR analysis in the MT pericarp.

**Conclusions:**

This study has provided a global picture of the gene expression changes in a novel mutant with distinct color in the fruit pericarp of pummelo. Interpretation of differentially expressed genes (DEGs) revealed new insight into the molecular regulation of β-carotene accumulation in the MT pericarp.

**Electronic supplementary material:**

The online version of this article (doi:10.1186/s12870-015-0435-3) contains supplementary material, which is available to authorized users.

## Background

Citrus is one of the most important fruit crops with great economic significance and value for humans in the world [1]. As a crucial quality feature, the external colour of citrus fruit first attracts the attention of consumers, and uniform bright coloration will enhance the fruit attractiveness and consumers’ acceptance. The external and internal coloration of most citrus fruits is due to the accumulation of carotenoids [2].

Carotenoids play indispensable roles in plants as components for all photosynthetic organisms and protectors against oxidation by quenching triplet chlorophyll, singlet oxygen, and superoxide anion radicals [3]. In higher plants, carotenoids provide flowers and fruits with distinct colors, ranging from yellow to orange or red, to attract insects and animals for pollination as well as seed dispersal [4,5]. Carotenoids also serve as precursors of the phytohormones abscisic acid (ABA), strigolactones, and other signalling molecules [6-8]. Some carotenoids are the precursors of vitamin A that cannot be artificially synthesized and therefore are essential nutritional components for animals and humans [9]. Moreover, they also have beneficial effects on human health, including enhancement of the immune system and reduction of the risk for degenerative diseases such as cancer, cardiovascular diseases and cataract [10-12]. Today, carotenoids are extensively used in health and nutritional products as important micronutrients [10].

Carotenoids are naturally synthesized in chloroplasts and chromoplasts by enzymes that are nuclear encoded [13]. In higher plants, structural genes of the carotenoid biosynthesis pathway have been isolated and characterized [14-18]. The first committed step of carotenoid biosynthesis is a head-to-head condensation of two molecules of a C20 precursor, geranylgeranyl pyrophosphate (*GGPP*), to form colourless phytoene catalyzed by the phytoene synthase (*PSY*). Next, the colourless phytoene is converted into the red lycopene by four desaturation reactions (catalyzed by phytoene desaturase, *PDS*, and ζ-carotene desaturase, *ZDS*) and (or) by two isomerization reactions mediated by carotene isomerase (*CRTISO*) and 15-cis-ζ-carotene isomerase (*ZISO*). Then, the lycopene flux branches into two pathways via cyclization reaction. Lycopene β-cyclase (*LCYB*) adds two β-rings to the ends of lycopene molecule to form β-carotene, while the co-action of *LCYb* and lycopene ε-cyclase (*LCYe*) generates α-carotene with one β-ring and one ε-ring. Subsequently, α-carotene is converted into lutein by hydroxylations catalyzed by ε-ring hydroxylase and β-ring hydroxylase (*BCH*). Then, zeaxanthin and violaxanthin are generated from β-carotene with hydroxylation reactions catalyzed by HYb and epoxydation catalyzed by zeaxanthin epoxidase (*ZEP*). The plant hormone ABA is an end product of the carotenoid biosynthetic pathway generated by the enzymatic cleavage of 9-cis-epoxycarotenoid dioxygenase (*NCEDs*). Carotenoid cleavage dioxygenases (*CCDs*) cleave carotenoids into apocarotenoids at different double-bond positions. In the last decade, due to the importance of carotenoids, many efforts have been made to understand the molecular basis of the regulation of carotenoid biosynthesis and accumulation.

Citrus is a complex source of carotenoids, with the largest number of carotenoid species found in any one fruit [19]. More than 115 different carotenoids have been identified in the pericarp and pulp of citrus, including lycopene, β-carotene, β-cryptoxanthin, zeaxanthin, and violaxanthin [20]. Because of the large diversity of carotenoid patterns, citrus has become an important model species for studies on plant carotenoid metabolism [19,21], such as the analyses of carotenoid composition and content, and expression of the main carotenoid biosynthetic genes [22-26]. Mutants with alteration in the carotenoid biosynthetic pathway have proven to be useful experimental materials for identifying molecular mechanisms regulating the process [27]. In the past few years, many pulp mutants have been identified in grapefruit (*Citrus paradisi*) and orange (*Citrus sinensis*), such as Red marsh, Shara, Cara Cara, and Hong Anliu [28-32], and these mutants have been used to study the complex regulatory mechanism of carotenoid biosynthesis at the gene and/or protein expression level [33-37], facilitating the understanding of the carotenoid regulation mechanism in the pulp of citrus [38-41]. Due to the lack of mutants affected in the pericarp, the carotenoid regulation mechanism was less studied in pericarp compared with the pulp of citrus. Recently, an *orange-pericarp* mutant (MT) originating from Guanxi pummelo has been discovered in China and provided us a potential material for studying this regulation mechanism.

In this study, we investigated the composition and level of carotenoids and the expression of carotenoid biosynthetic genes in the pericarps of MT and wild type (WT) in the ripe stage. From the whole genome perspective, the differentially expressed genes (DEGs) in MT and WT were identified using the RNA-seq technology. The identified genes provide useful information for studying the molecular mechanism of carotenoid biosynthesis in citrus pericarp.

## Results

### β-carotene is significantly accumulated in the MT

**Figure 1:**
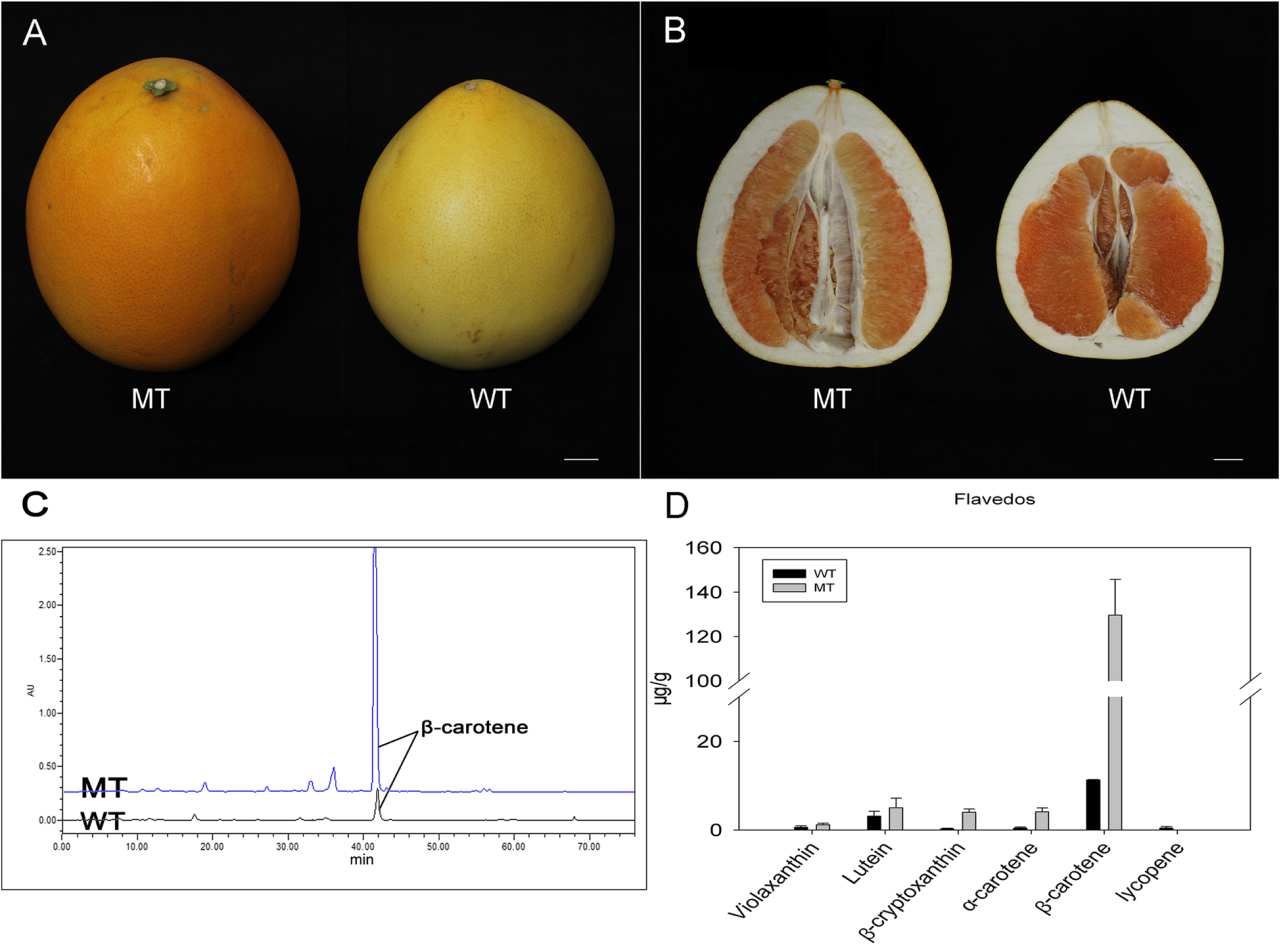
**The phenotype and carotenoid content in the WT and MT. (A, B)** Appearances of MT and WT fruits at maturation. **(C, D)** Carotenoid profiles and concentrations in the pericarps of WT and MT at fruit maturation. The bar represents 2 cm.

The pummelo MT was originally found in an orchard in Zhangzhou (Fujian, China) in the 2010s as a spontaneous bud mutation from the commercial variety of ‘guanxi’ pummelo. An obvious phenotypic change of the MT is the orange colour of the pericarp, showing a sharp contrast with the slight yellow colour of the mature pericarp in the WT fruit (Figure 1A, B). The *orange-pericarp* mutant was propagated by grafting onto different rootstocks and retained the stable phenotype of the orange-colour pericarp under field conditions, and no reversion to the parental phenotype has been observed so far. Moreover, 73 pairs of Simple Sequence Repeat (SSR) markers were used to evaluate the genetic background of the mutant. All the SSR patterns were the same between MT and WT (Additional file 1), indicating that the two genotypes shared an identical genetic background.

To characterize the phenotype differences between MT and WT, the carotenoid composition and content of mature fruits were analysed by High Performance Liquid Chromatography (HPLC). The most obvious difference in carotenoid between MT and WT pericarps was β-carotene content (Figure 1C, D). The β-carotene content of MT was about 10.5-fold that of the WT, accounting for 90.0% of the total identified carotenoids in MT. Additionally, the total carotenoid concentration of MT was 7.9-fold that of WT. Moreover, the concentrations of lutein, violaxanthin, α-carotene and β-cryptoxanthin were higher in MT than in WT. However, in the MT and WT pulps, the carotenoid species and content were similar to each other (Additional file 2).

### Three carotenogenic genes involved in β-carotene degradation are significantly down-regulated in the MT

**Figure 2:**
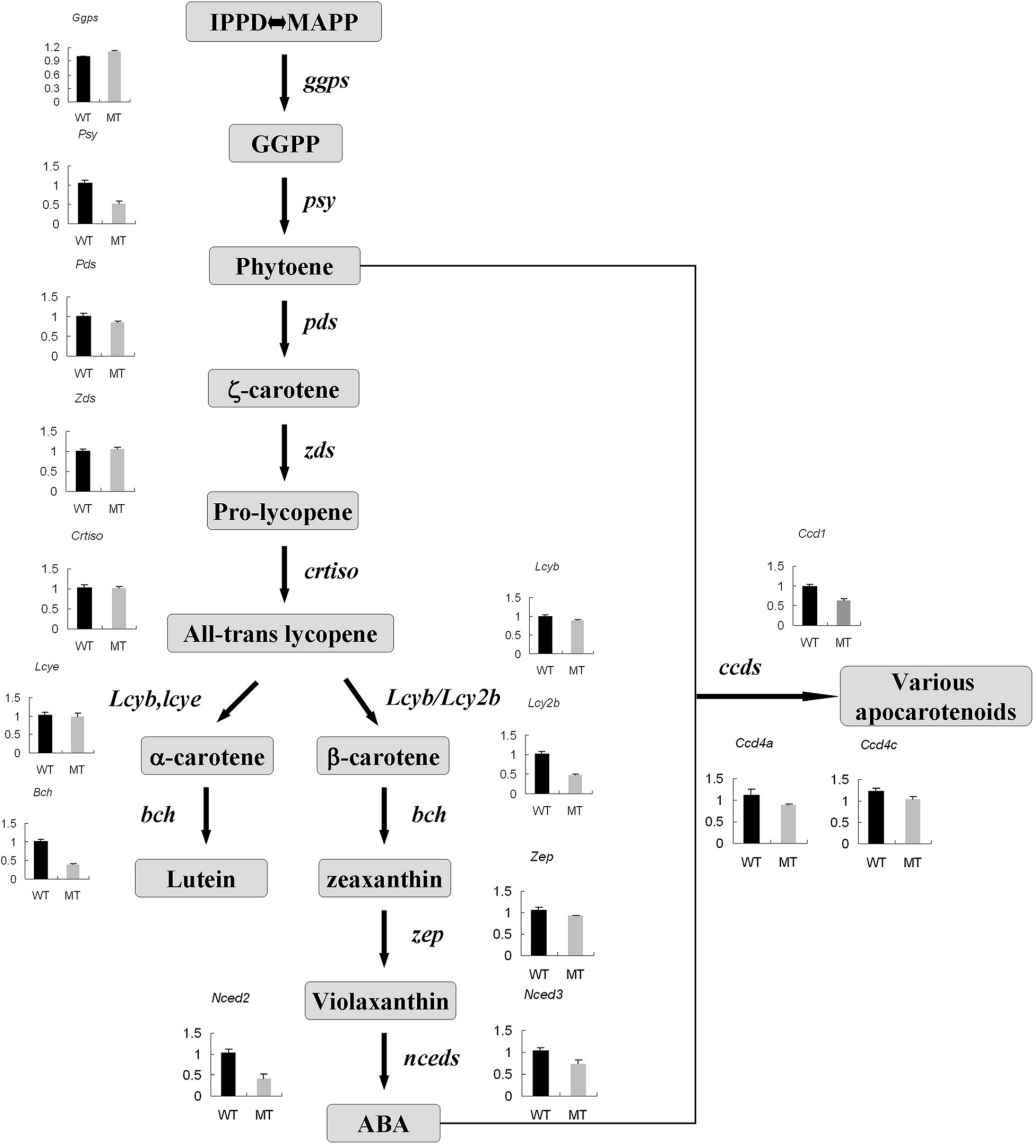
**Expression of carotenogenic genes in the pericarps of WT and MT at fruit maturation.**

Firstly, we compared the sequence information of the carotenoid biosynthetic genes in MT and WT and isolated full-length cDNAs, including *ggps*, *psy*, *pds*, *crtiso*, *lcyb*, *lcye*, *lcy2b*, *ccd4c*, *bch*, *nced2* and *nced3*. The result showed that the sequences were 100% identical between MT and WT (Additional file 3). These 11 sequence data were submitted to the GenBank with accession numbers from KP462725 to KP462735. Then, the effect of the mutation on carotenogenic gene expression was examined by quantitative real-time PCR (qRT-PCR) using the probes of pummelo cDNAs encoding GGPS, PSY, PDS, ZDS, CRTISO, LCYb, LCYe, LCY2b, CCD1, CCD4a, CCD4c, BCH, NCED2, NCED3 and ZEP (Figure 2). The expression levels of upstream carotenogenic genes (*ggps*, *zds* and *crtiso*) in MT and WT were almost the same. However, the gene expression level of *psy*, *pds* and *lcy2b* was much higher in WT than in MT. The expression level of all downstream carotenogenic genes was lower in MT than in WT. Particularly, *ccd1*, *bch* and *nced2* showed significantly reduced transcript levels in the MT pericarp.

### RNA-seq and global detection of DEGs

Solexa/Illumina RNA-Seq analysis was performed to identify the genes involved in the regulation of carotenoid biosynthesis in pummelo pericarp. Six libraries were constructed and sequenced, including three biological replicates for WT (termed as WT1, WT2 and WT3) and three biological replicates for MT (termed as MT1, MT2 and MT3). The major characteristics of these six libraries are summarized in Table 1. A sequencing depth of over thirteen million raw tags was obtained for each of the six libraries, with the number of raw tags ranging from 13,520,581 to 16,301,802. After filtration, we obtained a total of 13,347,784 (WT1), 14,532,229 (WT2) and 15,027,468 (WT3) clean tags for the WT RNA-Seq libraries and 16084513 (MT1), 14223118 (MT2) and 14025066 (MT3) clean tags for the MT RNA-Seq libraries, with the clean tags accounting for more than 98% of the total, which were then mapped to the sweet orange genome [42]. These reads were deposited in NCBI GEO database with an accession no. GSE64764. In the MT and WT samples, 76.0% (MT1), 76.5% (MT2), 76.4% (MT3), 75.9% (MT1), 76.4% (WT2) and 75.4% (WT3) of the clean tags from RNA-Seq data were mapped uniquely to the genome, while a small proportion of them were mapped multiply to the genome (Table 2)

**Table 1:** **Summary of sequence assembly after Illumina sequencing**

**Sample name**	**Raw reads**	**Clean reads**	**Clean bases**	**Error rate (%)**	**Q20 (%)**	**Q30 (%)**	**GC content (%)**
MT1	16301802	16084513	1.61G	0.03	97.25	91.76	43.5
MT2	14403818	14223118	1.42G	0.03	97.27	91.76	43.44
MT3	14197855	14025066	1.4G	0.03	97.31	91.87	43.53
WT1	13520581	13347784	1.33G	0.03	97.23	91.68	43.6
WT2	14715034	14532229	1.45G	0.03	97.29	91.82	43.49
WT3	15229307	15027468	1.5G	0.03	97.28	91.82	43.42

Q20: The percentage of bases with a Phred value > 20.

Q30: The percentage of bases with a Phred value > 30.

**Table 2:** S**ummary of clean reads mapped to the reference genome**

**Sample name**	**MT1**	**MT2**	**MT3**	**WT1**	**WT2**	**WT3**
Total reads	16084513	14223118	14025066	13347784	14532229	15027468
Total mapped	12712188 (79.03%)	11321201 (79.6%)	11196989 (79.84%)	10593747 (79.37%)	11554004 (79.51%)	11856432 (78.9%)
Multiple mapped	492073 (3.06%)	437971 (3.08%)	477365 (3.4%)	466493 (3.49%)	455224 (3.13%)	532109 (3.54%)
Uniquely mapped	12220115 (75.97%)	10883230 (76.52%)	10719624 (76.43%)	10127254 (75.87%)	11098780 (76.37%)	11324323 (75.36%)
Non-splice reads	8768392 (54.51%)	7846392 (55.17%)	7591808 (54.13%)	7219051 (54.08%)	7991617 (54.99%)	8176531 (54.41%)
Splice reads	3451723 (21.46%)	3036838 (21.35%)	3127816 (22.3%)	2908203 (21.79%)	3107163 (21.38%)	3147792 (20.95%)

.

Differentially expressed tags in the samples were identified by calculating the number of unambiguous tags for each gene and then normalizing this to the number of reads per kilobase of exon model per million mapped reads (RPKM). All the uniquely mapped reads were used for calculating the RPKM values of the genes. Genes within the RPKM range of 0–3 were considered to be expressed at low level; genes within the RPKM range of 3–15 were considered to be expressed at medium level; and genes beyond a RPKM value of 15 were considered to be expressed at high level [43]. Low-level expressed genes covered the highest percentage in MT and WT. The DEGs in the MT samples were identified at padj < 0.05, obtaining a total of 303 significantly DEGs, with 135 up-regulated and 168 down-regulated (Additional file 4). The details of these genes are listed in Additional file 5.

### Annotation of DEGs in MT and WT

**Figure 3:**
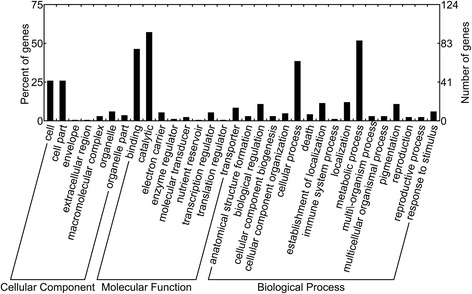
**Histogram of gene ontology classification.** The results are summarized in three main categories: molecular function, biological process and cellular component. The right Y-axis indicates the number of genes in a category. The left Y-axis indicates the percentage of a specific category of genes in that main category.

These DEGs may be involved in different functions. Gene ontology (GO) is an international standardized gene functional classification system that describes the properties of genes and their products in any organism. To understand the functions of the 303 DEGs, we mapped them to the three GO ontologies, including molecular function, cellular component, and biological process (Figure 3). According to cellular component, the most abundant DEGs were involved in “membrane” (9.2%), “cell” (5.3%) and “cell part” (5.3%). From the perspective of biological process, the DEGs were involved in “metabolic process” (28.4%), “cellular process” (20.8%), “organic substance metabolic process” (18.5%), “primary metabolic process” (17.8%) and “cellular metabolic process” (13.9%). In terms of molecular function, the genes were dominant in “catalytic activity” (31.4%), “binding” (24.4%), “ion binding” (15.5%), “heterocyclic compound binding” (13.5%) and “organic cyclic compound binding” (13.5%). In addition, the whole genome background was examined by GO category enrichment analysis (P-value ≤ 0.05). Three cellular component terms were significantly enriched in the up-regulated genes, including microtubule cytoskeleton, cytoskeletal part and cytoskeleton. To further understand the biological functions of these genes, KEGG (http://www.genome.jp/kegg/) ontology assignments were used to classify their functional annotations. All the 303 DEGs were assigned to 52 KEGG pathways. Among the pathways, carbon metabolism, starch and sucrose metabolism, biosynthesis of amino acids, and a few others were highly represented (Table 3).

**Table 3:** **Important KEGG pathways with more than 3 DEGs**

**KEGG pathway**	**Sample number**	**Gene ID**
Carbon metabolism	5	Serine hydroxymethyltransferase, Cysteine synthase, L-3-cyanoalanine synthase 2, Glyceraldehyde-3-phosphate dehydrogenase A, D-3-phosphoglycerate dehydrogenase
Starch and sucrose metabolism	4	Pectinesterase 3, sucrose-phosphate synthase 4, Pectinesterase 2, Alpha-1,4 glucan phosphorylase L-1 isozyme
Biosynthesis of amino acids	4	Serine hydroxymethyltransferase, Cysteine synthase, L-3-cyanoalanine synthase 2, D-3-phosphoglycerate dehydrogenase
Cyanoamino acid metabolism	3	Serine hydroxymethyltransferase, L-3-cyanoalanine synthase 2, Gamma-glutamyltranspeptidase 3
Pentose and glucuronate interconversions	3	Pectate lyase 5, Pectinesterase 3, Pectinesterase 2
Phagosome	3	Tubulin beta-1 chain, Tubulin alpha chain, Tubulin alpha chain, Tubulin beta-4 chain
Cysteine and methionine metabolism	3	Cysteine synthase, L-3-cyanoalanine synthase 2, 1-aminocyclopropane-1-carboxylate synthase

### Verification of DEGs

**Figure 4:**
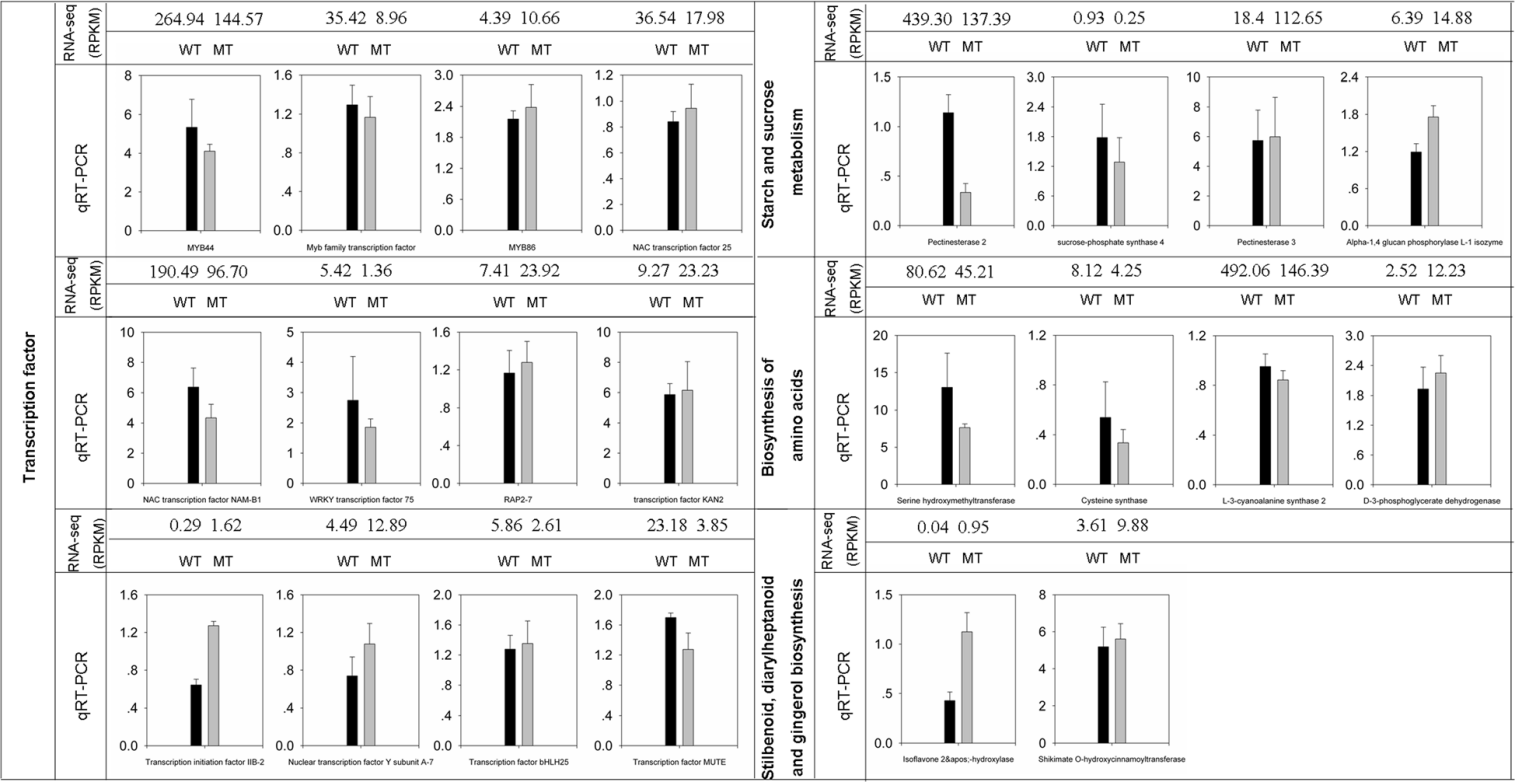
**RT-PCR analyses of differentially expressed genes corresponding to metabolic pathways and transcription factors in MT and WT.** The transcript abundance from RNA-seq data was added on the top of each gene. RPKM, reads per kilo bases per million reads.

A total of 22 DEGs were selected for qRT-PCR verification. Among them, 10 were referred to as the differentially expressed transcription factors. The other 12 genes belonged to the affected pathways including sugar metabolism and amino acid metabolism. The results showed that 19 out of the 22 differentially expressed genes in MT and WT were in consistency with the RNA-seq data (Figure 4). Linear regression [(RNA-seq value) = a(qRT-PCR value) + b] analysis of these 19 DEGs showed an overall correlation coefficient of 0.78, indicating a good correlation between the transcription profile revealed by RNA-seq data and the transcript abundance assayed by qRT-PCR (Additional file 6). These results confirmed the reliability of the RNA-seq data.

### Changes in fruit soluble sugar, amino acid, and fatty acid content

Considering the singificant expression change in a number of MT genes implicated in starch and sucrose metabolism as well as the biosynthesis of amino acids and fatty acids, the content of these metabolites was determined by the GC-MS analysis (Table 4). The results showed that the content of most sugars in MT was lower than that in WT, such as sucrose, glucose, fructose and mannose. Additionally, the MT pericarp, when compared with the WT pericarp, showed a decrease in the levels of four types of amino acids (proline, serine, threonine and GABA), but an increase in the levels of another four types of amino acids (lysine, valine, asparagine and aspartic acid). Interestingly, we detected an amount of asparagine in MT but trace in WT. We also detected four fatty acids in WT and MT pericarps. The content of octadecanoic acid and hexadecanoic acid was significantly lower in the MT pericarp than in the WT pericarp.

**Table 4:** **Accumulated sugars, amino acids and fatty acids in MT and WT pericarps**

		**MT (mg/g)**	**WT(mg/g)**
**Sugars**	Sucrose	5.048 ± 0.293	5.489 ± 0.255
	Glucose	0.306 ± 0.042	0.662 ± 0.024
	Fructose	1.046 ± 0.103	2.006 ± 0.021
	Mannose	1.122 ± 0.087	3.186 ± 0.234
	Glucopyranose	0.009 ± 0.002	0.009 ± 0.003
	Fructofuranose	0.231 ± 0.012	0.721 ± 0.080
	Talofuranose	0.476 ± 0.173	1.266 ± 0.149
	Xylose	0.013 ± 0.0004	0.024 ± 0.001
	4-Keto-glucose	0.009 ± 0.001	0.014 ± 0.0004
**Amino acids**	Valine	0.018 ± 0.004	0.016 ± 0.004
	Proline	0.111 ± 0.013	0.143 ± 0.061
	Serine	0.035 ± 0.011	0.046 ± 0.010
	Threonine	Trace	0.010 ± 0.006
	Lysine	0.027 ± 0.012	0.024 ± 0.006
	Aspartic acid	0.006 ± 0.001	Trace
	GABA	0.014 ± 0.006	0.016 ± 0.007
	Asparagine	0.760 ± 0.247	Trace
**Fatty acids**	Octadecanoic acid	0.255 ± 0.132	0.441 ± 0.073
	Hexadecanoic acid	0.504 ± 0.130	0.767 ± 0.073
	Octadecanoic acid,2,3-bisoxypropylester	0.035 ± 0.004	0.043 ± 0.006
	Hexadecanoic acid,2,3-bisoxypropylester	0.089 ± 0.019	0.098 ± 0.004

## Discussion

The mutant used in this study is derived from a spontaneous mutation in Guanxi pummel, and the mutation confers a novel phenotype that is regulated in a fruit-specific pattern, with the pericarp exhibiting obvious orange colour. The distinctive orange colour in the mutant pericarp has clearly been shown to be due to the massive accumulation of β-carotene. The β-carotene accumulation induced by the mutation also leads to an obvious increase of total carotenoids in the MT. In the past few years, many citrus carotenoid mutants have been discovered, but almost all of them show the red-fleshed phenotype and have proved to accumulate abnormal lycopene. Therefore, the pummelo MT identified in this study is a special material for the citrus carotenoid regulation study, particularly for the investigation of pigmentation regulation in pericarp. Previous studies on carotenoid biosynthesis in red-fleshed mutant concluded that the induction of lycopene accumulation coincided with increased expression of upstream carotenogenic genes and reduced expression of genes downstream of lycopene synthesis [30]. We hypothesized that the mechanism regulating the β-carotene accumulation was coincident with that of lycopene. As expected, the downstream genes of β-carotene degradation in the carotenoid biosynthetic pathway (*ccd1*, *ccd4a*, *ccd4c*, *bch*, *nced2*, *nced3* and *zep*) exhibited a decreased expression level in MT. Previous studies in potato tubers found that silencing the *bch* gene can significantly enhance β-carotene levels [44,45]. In maize, the *bch* alleles associated with reduced transcript expression also correlate with higher β-carotene concentrations [46]. In our research, the expression of *bch* in the WT was 1.58 fold that of the MT, indicating that the significantly reduced expression of *bch* may result in the amount accumulation of β-carotene in the MT pericarp. However, our analyses failed to find a dramatic increased expression of upstream carotenogenic genes in MT when compared with WT. Three key enzymes (*psy*, *lcy2b* and *lcyb*) for the β-carotene accumulation exhibited an obvious decrease in MT expression. These results implied that the MT exerted a major effect on β-carotene accumulation via the down-regulation of downstream genes, especially *bch*.

To understand the potential mechanisms involved in the regulation of carotenoid biosynthesis in the citrus pericarp, we used the RNA-seq approach to investigate the transcriptome profiles in MT and WT. Our analysis showed that a total of 303 genes altered expression pattern. Similar results have been reported in several studies on mutant–progenitor pairs [33,36,37]. GO analysis of annotated genes revealed that most of the DEGs were involved in catalytic activity and metabolic process (Figure 3). Because carotenoid biosynthesis which belonging to the secondary metabolisms is a dynamic and complex process catalyzed by a series of enzymes. Functional category analysis revealed that the DEGs are involved in a number of important pathways (Table 3), such as the metabolic pathways, which is consistent with the GO results that large numbers of genes are implicated in catalytic activity and metabolic process. The most noticeable pathways are carbon metabolism, starch and sucrose metabolism and biosynthesis of amino acids. Expressions of key genes in sucrose and starch metabolism, including alpha-1, 4 glucan phosphorylase (Cs6g22020), pectinesterase 3 (Cs1g16550), sucrose-phosphate synthase 4 (Cs5g19060) and pectinesterase 2 (orange1.1 t00214), were differentially expressed in WT and MT pericarpes, indicating that the sucrose and starch metabolism was significantly affected in MT. For example, Alpha-1, 4 glucan phosphorylase involved in sucrose degradation was up-regulated and sucrose-phosphate synthase 4 involved in sucrose accumulation was down-regulated in MT, indicating the acceleration of the sucrose degradation. Our gas chromatography–mass spectrometry (GC-MS) analysis also proved that the sucrose degradation in pericarp is higher in MT than in WT (Table 4). Moreover, the content of most sugars was significantly decreased in MT, indicating that the precursors for the glycolysis were increased by the accelerated degradation of sugars. Previous reports have also proved that the β-carotene synthesis was tightly linked to carbon metabolism [47,48]. Five genes involved in carbon metabolism were differentially expressed in MT and WT in our results. One gene encoding glyceraldehyde-3-phosphate dehydrogenase (Cs2g14940) was significantly increased (2.9-fold) in MT. This gene, catalyzing the conversion of glycerate 3-phosphate to glyceraldehyde 3-phosphate, was important for glycolysis, which was consistent with a previous speculation that glycolysis was increased in MT. The present research also found that five genes involved in amino acid biosynthesis were significantly changed in MT, which was in line with our GC-MS analysis that the content of amino acid differed significantly between MT and WT. A similar result was also observed in carotenoid-enhanced transgenic tomato fruits [49]. Interestingly, our research found that the asparagine was the most affected amino acid. Compared to WT, the content of asparagine increased 8.85-fold in the carotenoid-enhanced transgenic tomato fruits. These data indicated that the content of asparagine was strongly correlated with carotenoid accmulation.

In order to identify potential candidate genes involved in the regulation of carotenoid biosynthesis, we also analysed the top 10 most DEGs in MT and WT (Additional file 7). Among them, two genes were involved in fatty acid metabolism. One gene encoding Fatty acyl-CoA reductase 3 (Cs8g15220) was significantly reduced in the MT, which was important for the fatty acid biosynthesis. The other gene encoding GDSL esterase/lipase (Cs2g04220) was significantly increased in the MT, and the GDSL esterase/lipase was involved in fatty acid degradation. The altered expression of these two genes indicated a decrease of the fatty acid content in MT, which was consistent with our GC-MS analysis result that the contents of octadecanoic acid and hexadecanoic acid were lower in MT than in WT (Table 4). The biosynthesis of carotenoids and fatty acids required common precursors from pyruvate [50]. We concluded that these two genes may play important role in the carotenoid metabolism regulation. We also found that the expression of one gene belonging to cytochrome P450 (Cs6g20050) was significantly increased in MT. Cytochrome P450 catalyzes various reactions in plant biosynthesis of second metabolites, including carotenoids [51,52]. Cytochrome P450 hemoproteins, which catalyze NADPH- and O_2_-dependent hydroxylation reactions, were postulated to also be able to use hydrocarbon carotenes as substrates [53].

Transcription factors are the key switches for secondary metabolite gene regulation [54]. In the present study, twelve genes encoding transcription factors were identified by RNA-Seq analysis (Additional file 8). Among the group of transcription factors, we identified three genes belonging to the MYB family of transcription factors (Cs3g02020, Cs3g23070 and orange1.1 t01787). Previous studies on the carotenoid mutants also identified a number of MYB transcription factors [34,35]. The superfamily of MYB transcription factors was proved to control many biological processes, primarily in anthocyanin biosynthesis [55,56]. Overexpression of a *Vitis vinifera* R2R3-MYB transcription factor (*MYB5b*) in tomato resulted in an increased content of β-carotene [57]. These results indicated that the *MYB* genes may be involved in regulating carotenoid biosynthesis. We also detected two significantly differentially expressed NAC transcription factors. NAC proteins constitute one of the largest families of plant-specific transcription factors [58]. Genes from this family participate in various biological processes including developmental programs, defence, and biotic and abiotic stress responses [59,60]. Recently, a NAC transcription factor (SlNAC4) has been proved to a positive regulator of carotenoid accumulation [61]. In this study, both of the two identified NAC transcription factors showed a down-regulated expression in MT samples, indicating that both of them may play a feedback regulating role in the carotenoid biosynthesis. Ethylene plays a key regulatory role in fruit ripening and carotenoid accumulation [62]. Our results showed that the ethylene-responsive transcription factor (RAP2-7) was highly expressed in MT. In this study, we also identified several other significantly differentially expressed transcription factors, such as WRKY (Cs2g25560), BHLH (Cs8g03200) and MUTE (Cs9g06130).

## Conclusions

This is the first investigation of the biochemical and molecular alterations associated with an *orange-pericarp* fruit mutation in pummelo. In this study, the content of carotenoids and the expression patterns of carotenoid biosynthetic genes in the pericarps were comparatively analysed for the pummelo MT and its WT. We used RNA-seq to identify the differential expression genes in the MT by comparing with the WT. GO analysis and pathway mapping of the DEGs provide significant insight into the underlying molecular mechanisms governing the β-carotene accumulation. Critical genes and pathways involved in carbon metabolism, starch and sucrose metabolism and biosynthesis of amino acids were associated with the β-carotene accumulation. The results suggest that the considerable β-carotene accumulation appears to be due to a down-regulation of downstream genes for β-carotene degradation. Moreover, several candidate genes and transcription factors that possibly regulate carotenoid biosynthesis in the pericarp of pummelo were also identified. However, the functions of these genes remain to be elucidated in the future. The overall findings from this study facilitate the understanding of the molecular regulation of β-carotene accumulation in the pummelo mutant strain and provide useful information for further related studies.

## Methods

### Plant materials and RNA extraction

The materials used in this study were ‘Guanxi’ pummelo and its MT cultivated in the city of Zhangzhou, Fujian province, China. The samples were harvested at ripe stage with three biological replicates. After separation from fruits, the pericarps were immediately frozen in liquid nitrogen and kept at −80°C until further use. Total RNA was extracted from the pericarps of WT and MT as previously described [30]. The quality of the RNA was assessed by 1% agarose gel electrophoresis coupled with NanoPhotometer® spectrophotometer (IMPLEN, CA, USA). RNA concentration was measured using Qubit® RNA Assay Kit in Qubit® 2.0 Flurometer (Life Technologies, USA). RNA integrity was confirmed using a 2100 Bioanalyzer (Agilent Technologies) with a minimum RNA integrity number (RIN) value of 8.0.

### Carotenoid content measurement

Carotenoid extraction and quantification was performed as previously described with modification [30]. Carotenoids were analyzed by reversed phase HPLC. Chromatography was carried out with a Waters liquid chromatography system equipped with a model 600E solvent delivery system, a model 2996 photodiode array detection (PAD) system, a model 717 plus autosampler, and an empower Chromatography Manager. Carotenoids were eluted with MeOH- Acetonitrile [75:25 v/v, eluent A] and MTBE [eluent B] using a C30 carotenoid column (15 × 4.6 mm; YMC, Japan). Carotenoids were identified by their characteristic absorption spectra, typical retention time, and comparison with authentic standards (Bern, Switzerland).

### RNA-seq library preparation and sequencing

Sequencing libraries were constructed by using three biological replicates for WT and MT pericarps, which were named WT1, WT2, WT3, MT1, MT2 and MT3, respectively. A total amount of 3 μg RNA per sample was used as input material for the RNA sample preparation. Sequencing libraries were generated using NEBNext® Ultra™ RNA Library Prep Kit for Illumina® (NEB, USA) by following manufacturer’s recommendations, and index codes were added to attribute sequences to each sample. Briefly, mRNA was purified from total RNA using poly-T oligo-attached magnetic beads. Fragmentation was carried out using divalent cations under elevated temperature in NEBNext First Strand Synthesis Reaction Buffer (5×). First strand cDNA was synthesized using random hexamer primer and MmuLV Reverse Transcriptase (RNase H-). Second strand cDNA synthesis was subsequently performed using DNA polymerase I and RNase H. Remaining overhangs were converted into blunt ends via exonuclease/polymerase activities. After adenylation of 3′ ends of DNA fragments, NEBNext Adaptor with hairpin loop structure was ligated before hybridization. To preferentially select cDNA fragments of 150–200 bp in length, the library fragments were purified with AMPure XP system (Beckman Coulter, Beverly, USA). Then 3 μl USER Enzyme (NEB, USA) was used with size-selected, adaptor-ligated cDNA at 37°C for 15 min followed by 5 min at 95°C before PCR. The PCR was performed with Phusion High-Fidelity DNA polymerase, Universal PCR primers and Index (X) Primer. Finally, PCR products were purified (AMPure XP system) and library quality was assessed on the Agilent Bioanalyzer 2100 system. The clustering of the index-coded samples was performed on a cBot Cluster Generation System using TruSeq SR Cluster Kit v3-cBot-HS (Illumia) according to the manufacturer’s instructions. After cluster generation, the library preparations were sequenced on an Illumina Hiseq 2000 platform and 100 bp single-end reads were generated.

### Data analysis

Raw sequence reads were first processed using an in-house Perl script. In this step, clean data were obtained by removing reads containing adaptors only, reads with more than 10% unknown bases and reads with a quality score of less than 5.0 for more than half of the bases. Meanwhile, the Q20, Q30 and GC content of the clean data were calculated. All the downstream analyses were based on these clean data with high quality. For annotation, all clean tags were mapped to the reference sequence of the sweet orange genome [42]. Mismatches of no more than two bases were allowed in the alignment. The remaining clean tags were designated as unambiguous clean tags. The RPKM method was used to estimate the unique gene expression levels [63]. Reference genome and gene model annotation files were downloaded directly from the genome website (http://citrus.hzau.edu.cn/orange/index.php). Index of the reference genome was built using Bowtie v2.0.6 (Broad Institute, Cambridge, MA, USA) and single-end clean reads were aligned to the reference genome using TopHat v2.0.9 (Broad Institute). TopHat was selected as the mapping tool because it can generate a database of splice junctions based on the gene model annotation file and thus give a better mapping result than other non-splice mapping tools. Differential expression analysis of two samples (each three biological replicates) was performed using the DESeq R package (1.10.1) [64]. DESeq provides statistical routines for determining differential expression in digital gene expression data using a model based on the negative binomial distribution. The resulting P-values were adjusted using the Benjamini and Hochberg’s approach for controlling the false discovery rate. The significance of the gene expression difference was determined with an adjusted P-value <0.05 found by DESeq. GO enrichment analysis of DEGs was implemented by the GOseq R package. GO terms with a corrected P-value < 0.05 were considered significantly enriched by differentially expressed genes. The statistical enrichment of the differential expression genes in KEGG pathways was tested using the KO-Based Annotation System (KOBAS) software.

### qRT-PCR analysis

To validate the RNA-Seq results and provide more information for the affected metabolic processes, 22 selected DEGs corresponding to the metabolic pathways and transcription factors were verified by qRT-PCR. Actin was amplified along with the target gene as an endogenous control to normalize expression between different samples. Primer sequences used for qRT-PCR are listed in Additional file 9. The samples collected from another year and different from the RNA-seq analysis were used for qRT-PCR validation. One μg of total RNA from each sample was used to synthesize the first strand cDNA using the PrimeScript Reverse Transcriptase Kit (TaKaRa) according to the protocol of the manufacturer. The qRT-PCR was carried out in an ABI PRISM® 9600 Sequence Detection System (Applied Biosystems) using SYBR Green Supermix according to the manufacturer’s instructions, under the thermal cycle conditions of an initial denaturation at 94°C for 10 min, followed by 40 cycles of 94°C for 15 s, 60°C for 31 s for annealing, and a final step of extension at 72°C for 7 min. The expression level of genes was calculated by the delta-delta-Ct method [65]. Each biological sample was examined in duplicate with two to three technical replicates.

### Determination of the sugar, amino acid and fatty acid content in the pericarp

The extraction and derivatization of sugars, amino acids and fatty acids were performed as previously described with modification [66]. A 200 mg sample was added to the extracting solution containing 2,700 μl of methanol and 300 μl of 0.2 mg ml^−1^ ribitol in water as a quantification internal standard. Each sample (1 μl) was injected into the GC system through a fused-silica capillary column with a DB-5 MS stationary phase (30 m × 0.25 mm i.d., 0.25 μm). Total ion current (TIC) spectra were recorded in the mass range of 45–600 atomic mass units (amu) in scanning mode.

## Availability of supporting data

Raw sequencing data is available through the NCBI Gene Expression Omnibus under Project ID GSE64764. All samples were sequenced as 100 bp single reads on an Illumina HiSeq2500 sequencer.

## Additional files

Additional file 1:
**SSR marker analysis of MT and WT.** For each pair of SSR markers, the left is WT, and the right is MT. Additional file 2:
**Carotenoid content in the pulps of MT and WT at fruit maturation.**
Additional file 3:
**Sequence information of carotenoid biosynthetic genes in MT and WT.**
Additional file 4:
**DEGs in MT and WT.** The red part represents the genes up-regulated in MT as compared to WT. The green part shows the genes downregulated in MT. The blue part shows the genes without expression difference between the two samples. Additional file 5:
**List of significantly DEGs between MT and WT.**
Additional file 6:
**Comparison of gene expression ratios observed by RNA-seq and qRT-PCR.** The RNA-seq log_2_ (expression ratio) values (x-axis) are plotted against the log_2_ (expression ratio) obtained by qRT-PCR (y-axis). Additional file 7:
**Top 10 most DEGs in MT and WT.** The transcript abundance from RNA-seq data was added on the top of each gene. RPKM, reads per kilo bases per million reads. The gene number refers to the sweet orange genome. Additional file 8:
**Transcription factor with altered expression in MT.**
Additional file 9:
**Primer sequences for amplification by qRT-PCR.**

## Abbreviations

HPLC: High Performance Liquid Chromatography
MT: *Orange-pericarp* mutant
WT: Wild type
qRT-PCR: Quantitative real-time PCR
GO: Gene ontology
DEGs: Differentially expressed genes
ABA: Abscisic acid
*GGPP*: Geranylgeranyl pyrophosphate
*PSY*: Phytoene synthase
*PDS*: Phytoene desaturase
*ZDS*: ζ-carotene desaturase
*CRTISO*: Carotene isomerase
*ZISO*: 15-cis-ζ-carotene isomerase
*LCYb*: Lycopene β-cyclase
*LCYe*: Lycopene ε-cyclase
*BCH*: β-ring hydroxylase
*ZEP*: Zeaxanthin epoxidase
*NCEDs*: 9-cis-epoxycarotenoid dioxygenase
*CCDs*: Carotenoid cleavage dioxygenases
SSR: Simple Sequence Repeat
RPKM: Reads per kilobase of exon model per million mapped reads
GC-MS: Gas chromatography–mass spectrometry
RIN: RNA integrity number
PAD: Photodiode array detection
TIC: Total ion current

## References

[1] Talon M, Gmitter FG (2008). Citrus genomics. Int J Plant Genomics.

[2] Alquézar B, Rodrigo M, Zacarías L (2008). Carotenoid biosynthesis and their regulation in citrus fruits. Tree Forestry Sci Biotechnol.

[3] Demmig-Adams B, Adams WW (2002). Antioxidants in photosynthesis and human nutrition. Science.

[4] Ma J, Li J, Zhao J, Zhou H, Ren F, Wang L (2014). Inactivation of a gene encoding carotenoid cleavage dioxygenase (CCD4) leads to carotenoid-based yellow coloration of fruit flesh and leaf midvein in peach. Plant Mol Biol Report.

[5] Rubio A, Rambla JL, Santaella M, Gómez MD, Orzaez D, Granell A (2008). Cytosolic and plastoglobule-targeted carotenoid dioxygenases from Crocus sativus are both involved in β-ionone release. J Biol Chem.

[6] Cazzonelli CI (2011). Goldacre review: carotenoids in nature: insights from plants and beyond. Funct Plant Biol.

[7] Fester T, Hause B, Schmidt D, Halfmann K, Schmidt J, Wray V (2002). Occurrence and localization of apocarotenoids in arbuscular mycorrhizal plant roots. Plant Cell Physiol.

[8] Milborrow B (2001). The pathway of biosynthesis of abscisic acid in vascular plants: a review of the present state of knowledge of ABA biosynthesis. J Exp Bot.

[9] Wolf G (2001). The discovery of the visual function of vitamin A. J Nutr.

[10] Krinsky NI, Johnson EJ (2005). Carotenoid actions and their relation to health and disease. Mol Asp Med.

[11] Rao A, Rao LG (2007). Carotenoids and human health. Pharmacol Res.

[12] Von Lintig J (2010). Colors with functions: elucidating the biochemical and molecular basis of carotenoid metabolism. Annu Rev Nutr.

[13] Sandmann G (2001). Carotenoid biosynthesis and biotechnological application. Arch Biochem Biophys.

[14] Cunningham F, Gantt E (1998). Genes and enzymes of carotenoid biosynthesis in plants. Annu Rev Plant Biol.

[15] Hirschberg J (2001). Carotenoid biosynthesis in flowering plants. Curr Opin Plant Biol.

[16] Park H, Kreunen SS, Cuttriss AJ, DellaPenna D, Pogson BJ (2002). Identification of the carotenoid isomerase provides insight into carotenoid biosynthesis, prolamellar body formation, and photomorphogenesis. Plant Cell Online.

[17] Ronen G, Cohen M, Zamir D, Hirschberg J (1999). Regulation of carotenoid biosynthesis during tomato fruit development: expression of the gene for lycopene epsilon-cyclase is down-regulated during ripening and is elevated in the mutantDelta. Plant J.

[18] Tanaka Y, Sasaki N, Ohmiya A (2008). Biosynthesis of plant pigments: anthocyanins, betalains and carotenoids. Plant J.

[19] Gross J, Schweigert BS (1987). Pigments in fruits. Food science and technology: a series of monographs.

[20] Saunt J (2000). Citrus varieties of the world.

[21] Rouseff R, Raley L, Hofsommer H-J (1996). Application of diode array detection with a C-30 reversed phase column for the separation and identification of saponified orange juice carotenoids. J Agric Food Chem.

[22] Sun Y, Liu D, Chen J, Ye X, Yu D (2011). Effects of different factors of ultrasound treatment on the extraction yield of the all-trans-β-carotene from citrus peels. Ultrason Sonochem.

[23] Zhou JY, Sun CD, Zhang LL, Dai X, Xu CJ, Chen KS (2010). Preferential accumulation of orange-colored carotenoids in Ponkan (*Citrus reticulata*) fruit peel following postharvest application of ethylene or ethephon. Sci Hortic.

[24] Rodrigo MJ, Alquézar B, Alós E, Medina V, Carmona L, Bruno M (2013). A novel carotenoid cleavage activity involved in the biosynthesis of Citrus fruit-specific apocarotenoid pigments. J Exp Bot.

[25] Ma G, Zhang L, Matsuta A, Matsutani K, Yamawaki K, Yahata M (2013). Enzymatic formation of β-citraurin from β-cryptoxanthin and zeaxanthin by carotenoid cleavage Dioxygenase4 in the flavedo of citrus fruit. Plant Physiol.

[26] Rodrigo MJ, Marcos JF, Zacarías L (2004). Biochemical and molecular analysis of carotenoid biosynthesis in flavedo of orange (*Citrus sinensis* L.) during fruit development and maturation. J Agric Food Chem.

[27] Rodrigo MJ, Marcos JF, Alférez F, Mallent MD, Zacarías L (2003). Characterization of Pinalate, a novel Citrus sinensis mutant with a fruit-specific alteration that results in yellow pigmentation and decreased ABA content. J Exp Bot.

[28] Lee HS (2001). Characterization of carotenoids in juice of red navel orange (Cara Cara). J Agric Food Chem.

[29] Monselise S, Halevy A (1961). Detection of lycopene in pink orange fruit. Science.

[30] Liu Q, Xu J, Liu YZ, Zhao XL, Deng XX, Guo LL (2007). A novel bud mutation that confers abnormal patterns of lycopene accumulation in sweet orange fruit (*Citrus sinensis* L. Osbeck). J Exp Bot.

[31] Curl AL, Bailey GF (1957). The carotenoids of Ruby Red grapefruit. J Food Sci.

[32] Xu J, Tao NG, Liu Q, Deng XX (2006). Presence of diverse ratios of lycopene/β-carotene in five pink or red-fleshed citrus cultivars. Sci Hortic.

[33] Liu Q, Zhu AD, Chai LJ, Zhou WJ, Yu KQ, Ding J (2009). Transcriptome analysis of a spontaneous mutant in sweet orange [*Citrus sinensis* (L.) Osbeck] during fruit development. J Exp Bot.

[34] Pan ZY, Liu Q, Yun Z, Guan R, Zeng WF, Xu Q (2009). Comparative proteomics of a lycopene-accumulating mutant reveals the important role of oxidative stress on carotenogenesis in sweet orange (*Citrus sinensis* [L.] osbeck). Proteomics.

[35] Xu Q, Yu KQ, Zhu AD, Ye JL, Liu Q, Zhang JC (2009). Comparative transcripts profiling reveals new insight into molecular processes regulating lycopene accumulation in a sweet orange (*Citrus sinensis*) red-flesh mutant. BMC Genomics.

[36] Alós E, Roca M, Iglesias DJ, Mínguez-Mosquera MI, Damasceno CMB, Thannhauser TW (2008). An evaluation of the basis and consequences of a stay-green mutation in the navel negra citrus mutant using transcriptomic and proteomic profiling and metabolite analysis. Plant Physiol.

[37] Licciardello C, Russo MP, Recupero RG (2008). Identification of differentially expressed genes in the flesh of blood and common oranges. Tree Genetics Genomes.

[38] Fanciullino AL, Dhuique-Mayer C, Luro F, Morillon R, Ollitrault P (2007). Carotenoid biosynthetic pathway in the Citrus genus: number of copies and phylogenetic diversity of seven genes. J Agric Food Chem.

[39] Xu CJ, Fraser PD, Wang WJ, Bramley PM (2006). Differences in the carotenoid content of ordinary citrus and lycopene-accumulating mutants. J Agric Food Chem.

[40] Tao NG, Hu ZY, Liu Q, Xu J, Cheng YJ, Guo LL (2007). Expression of phytoene synthase gene (Psy) is enhanced during fruit ripening of Cara Cara navel orange (Citrus sinensis Osbeck). Plant Cell Rep.

[41] Alquezar B, Rodrigo MJ, Zacarías L (2008). Regulation of carotenoid biosynthesis during fruit maturation in the red-fleshed orange mutant Cara Cara. Phytochemistry.

[42] Xu Q, Chen LL, Ruan XA, Chen DJ, Zhu AD, Chen CL (2013). The draft genome of sweet orange (*Citrus sinensis*). Nat Genet.

[43] Zhang N, Zhang HJ, Zhao B, Sun QQ, Cao YY, Li R (2014). The RNA-seq approach to discriminate gene expression profiles in response to melatonin on cucumber lateral root formation. J Pineal Res.

[44] Van Eck J, Conlin B, Garvin D, Mason H, Navarre D, Brown C (2007). Enhancing beta-carotene content in potato by RNAi-mediated silencing of the beta-carotene hydroxylase gene. Am J Potato Res.

[45] Diretto G, Welsch R, Tavazza R, Mourgues F, Pizzichini D, Beyer P (2007). Silencing of beta-carotene hydroxylase increases total carotenoid and beta-carotene levels in potato tubers. BMC Plant Biol.

[46] Yan JB, Kandianis CB, Harjes CE, Bai L, Kim E-H, Yang X (2010). Rare genetic variation at Zea mays crtRB1 increases [beta]-carotene in maize grain. Nat Genet.

[47] Schulze-Siebert D, Schultz G (1987). β-carotene synthesis in isolated spinach chloroplasts its tight linkage to photosynthetic carbon metabolism. Plant Physiol.

[48] Schultz G, Heintze A, Hoppe P, Hagelstein P, Gorlach J, Meereis K (1991). Tocopherol and carotenoid synthesis in chloroplasts: tight linkage to plastidic carbon metabolism in developing chloroplasts. Am Soc Plant Physiol.

[49] Fraser PD, Enfissi EM, Halket JM, Truesdale MR, Yu D, Gerrish C (2007). Manipulation of phytoene levels in tomato fruit: effects on isoprenoids, plastids, and intermediary metabolism. Plant Cell Online.

[50] Schwender J, Seemann M, Lichtenthaler H, Rohmer M (1996). Biosynthesis of isoprenoids (carotenoids, sterols, prenyl side-chains of chlorophylls and plastoquinone) via a novel pyruvate/glyceraldehyde 3-phosphate non-mevalonate pathway in the green alga Scenedesmus obliquus. Biochem J.

[51] Kim J, DellaPenna D (2006). Defining the primary route for lutein synthesis in plants: the role of Arabidopsis carotenoid β-ring hydroxylase CYP97A3. Proc Natl Acad Sci U S A.

[52] Tian L, DellaPenna D (2001). Characterization of a second carotenoid β-hydroxylase gene from Arabidopsis and its relationship to the LUT1 locus. Plant Mol Biol.

[53] Chapple C (1998). Molecular-genetic analysis of plant cytochrome P450-dependent monooxygenases. Annu Rev Plant Biol.

[54] De Geyter N, Gholami A, Goormachtig S, Goossens A (2012). Transcriptional machineries in jasmonate-elicited plant secondary metabolism. Trends Plant Sci.

[55] Kobayashi S, Goto-Yamamoto N, Hirochika H (2004). Retrotransposon-induced mutations in grape skin color. Science.

[56] Takos AM, Jaffé FW, Jacob SR, Bogs J, Robinson SP, Walker AR (2006). Light-induced expression of a MYB gene regulates anthocyanin biosynthesis in red apples. Plant Physiol.

[57] Mahjoub A, Hernould M, Joubès J, Decendit A, Mars M, Barrieu F (2009). Overexpression of a grapevine R2R3-MYB factor in tomato affects vegetative development, flower morphology and flavonoid and terpenoid metabolism. Plant Physiol Biochem.

[58] Puranik S, Sahu PP, Srivastava PS, Prasad M (2012). NAC proteins: regulation and role in stress tolerance. Trends Plant Sci.

[59] Olsen AN, Ernst HA, Leggio LL, Skriver K (2005). NAC transcription factors: structurally distinct, functionally diverse. Trends Plant Sci.

[60] Hegedus D, Yu M, Baldwin D, Gruber M, Sharpe A, Parkin I (2003). Molecular characterization of Brassica napus NAC domain transcriptional activators induced in response to biotic and abiotic stress. Plant Mol Biol.

[61] Zhu M, Chen G, Zhou S, Tu Y, Wang Y, Dong T (2014). A new tomato NAC (NAM/ATAF1/2/CUC2) transcription factor, SlNAC4, functions as a positive regulator of fruit ripening and carotenoid accumulation. Plant Cell Physiol.

[62] Lee JM, Joung JG, McQuinn R, Chung MY, Fei Z, Tieman D (2012). Combined transcriptome, genetic diversity and metabolite profiling in tomato fruit reveals that the ethylene response factor SlERF6 plays an important role in ripening and carotenoid accumulation. Plant J.

[63] Mortazavi A, Williams BA, Mccue K, Schaeffer L, Wold B (2008). Mapping and quantifying mammalian transcriptomes by RNA-Seq. Nat Methods.

[64] Anders S, Huber W. Differential expression analysis for sequence count data. Genome Biol. 2010;11(10). 10.1186/gb-2010-11-10-r106 PMC3218662 20979621

[65] Livak KJ, Schmittgen TD (2001). Analysis of relative gene expression data using real-time quantitative PCR and the 2^− ΔΔCT^ method. Methods.

[66] Yun Z, Gao HJ, Liu P, Liu SZ, Luo T, Jin S (2013). Comparative proteomic and metabolomic profiling of citrus fruit with enhancement of disease resistance by postharvest heat treatment. BMC Plant Biol.

