# Supplementary figures and images for: Transcriptomic analysis of differentially expressed genes in an orange-pericarp mutant and wild type in pummelo (Citrus grandis)
Source: BMC Plant Biol. 2015 Feb 12;15:44. doi: 10.1186/s12870-015-0435-3 (PMC4352283; doi:10.1186/s12870-015-0435-3)

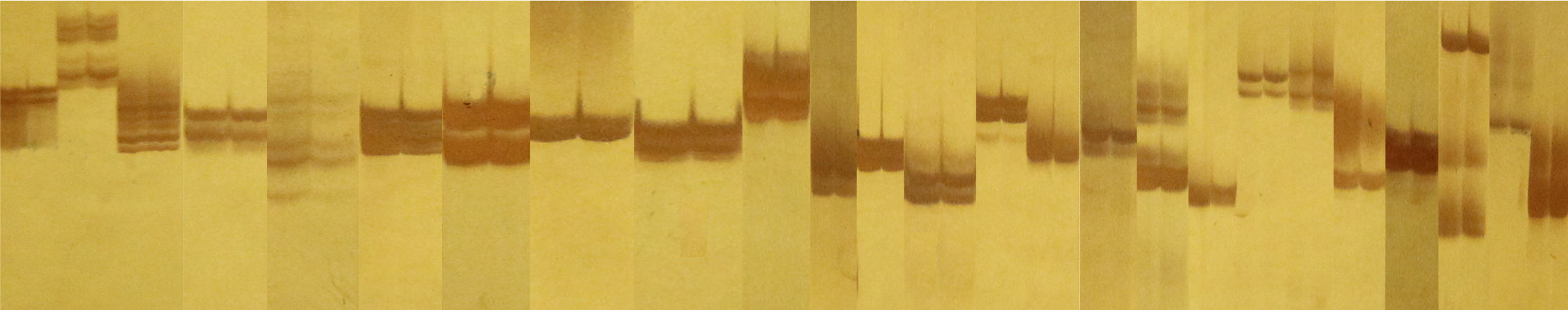

Supplement: Additional file 1: — SSR marker analysis of MT and WT. For each pair of SSR markers, the left is WT, and the right is MT. [file 12870_2015_435_MOESM1_ESM.tiff]

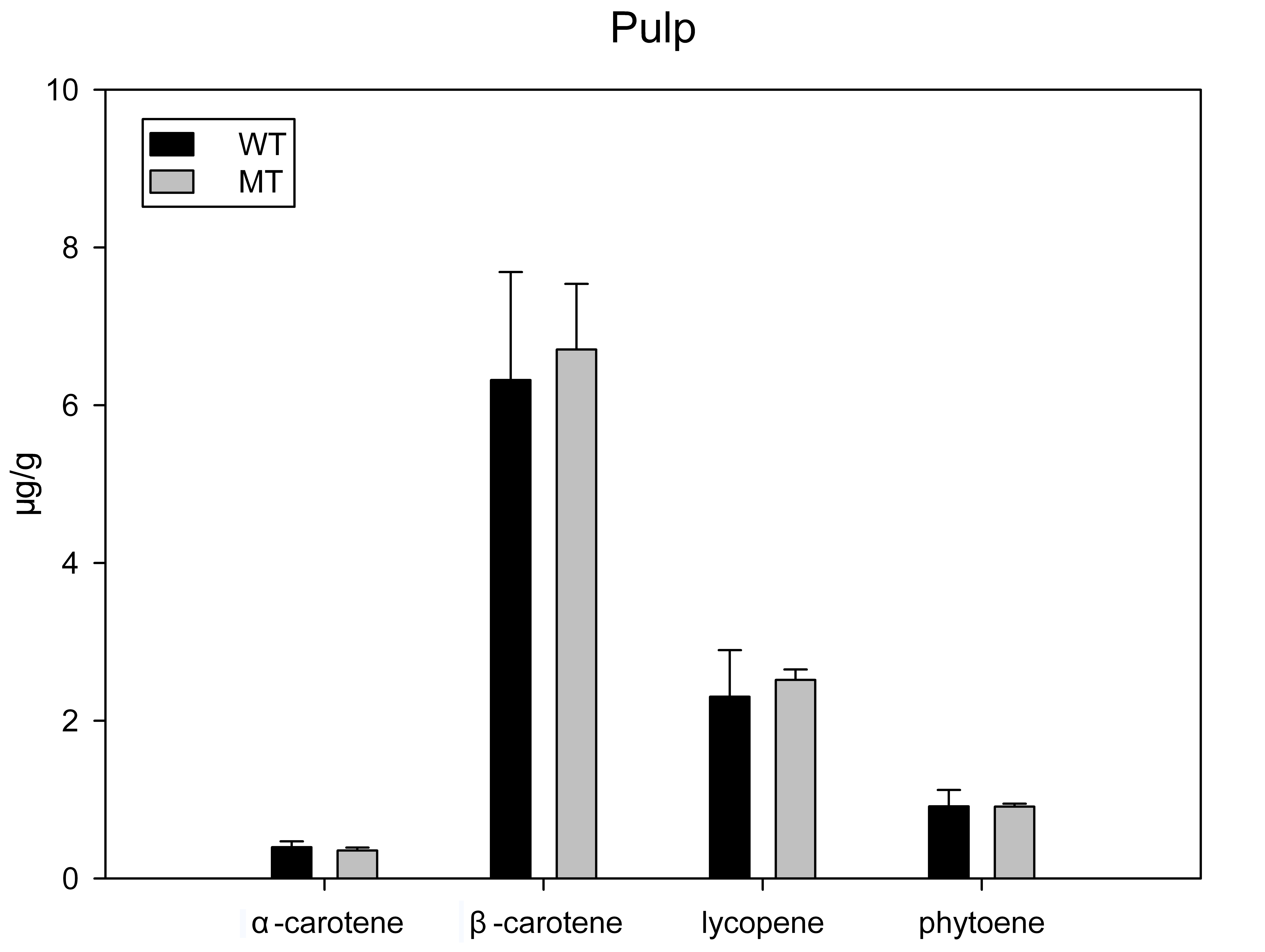

Supplement: Additional file 2: — Carotenoid content in the pulps of MT and WT at fruit maturation. [file 12870_2015_435_MOESM2_ESM.tiff]

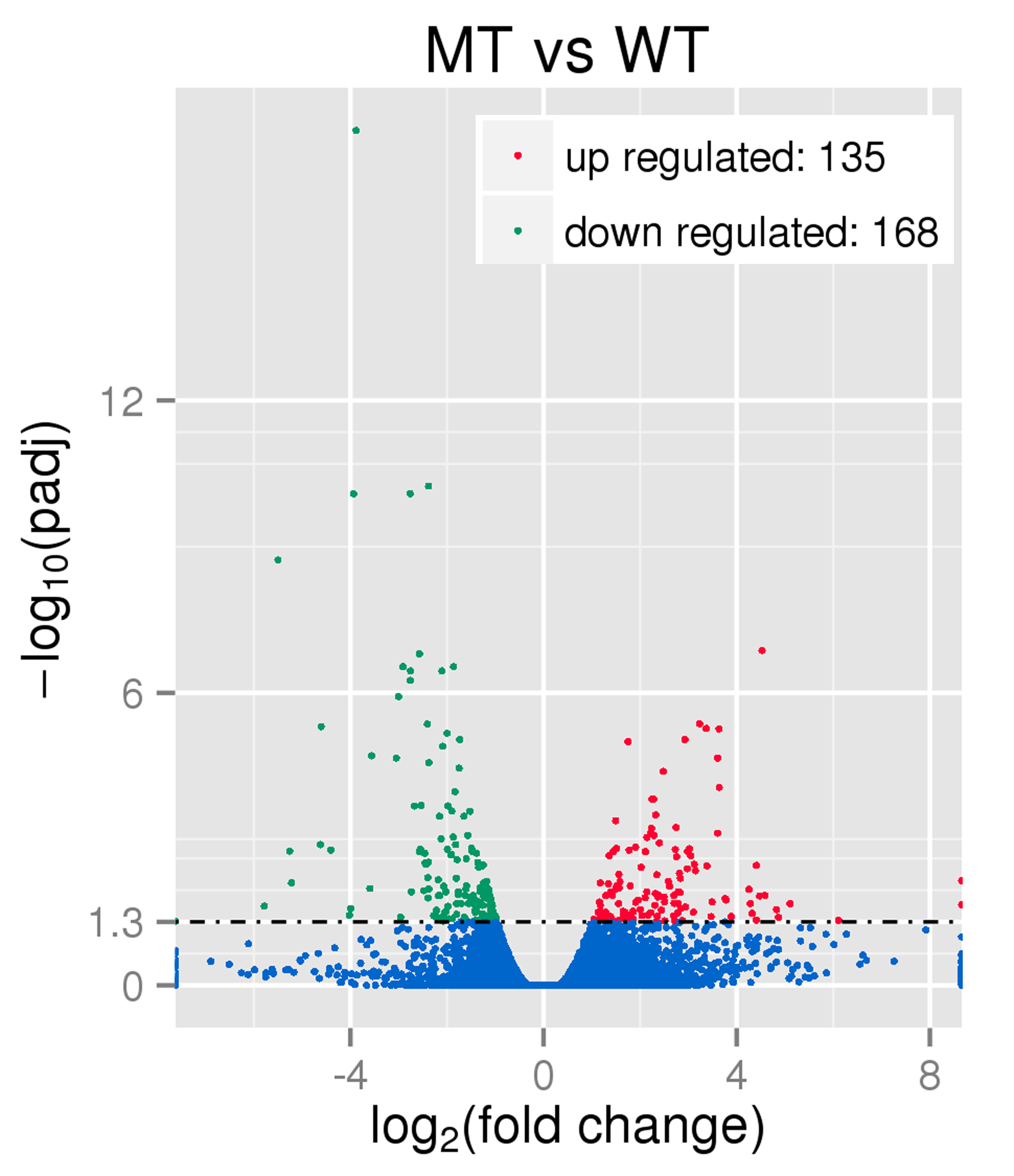

Supplement: Additional file 4: — DEGs in MT and WT. The red part represents the genes up-regulated in MT as compared to WT. The green part shows the genes downregulated in MT. The blue part shows the genes without expression difference between the two samples. [file 12870_2015_435_MOESM4_ESM.tiff]

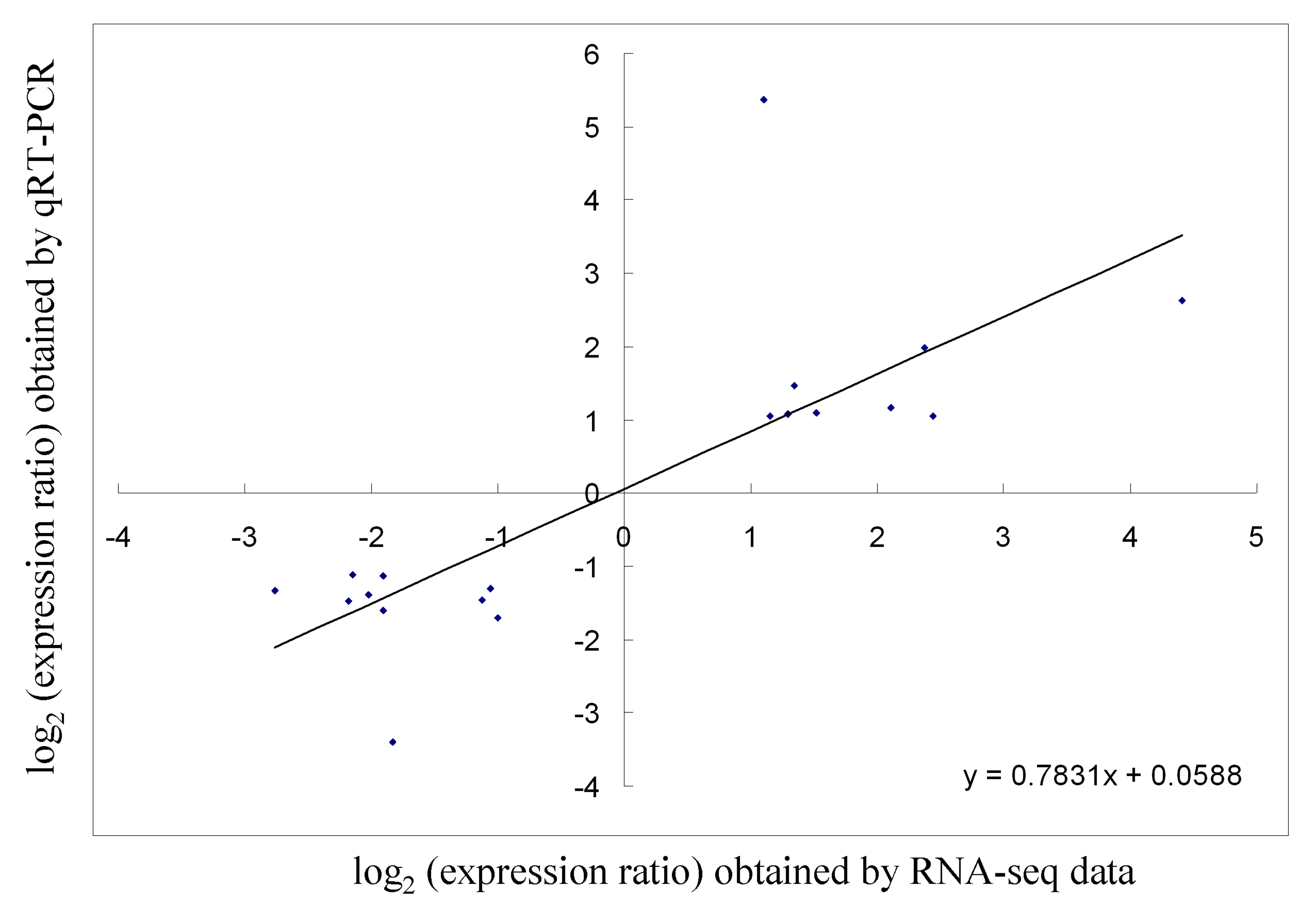

Supplement: Additional file 6: — Comparison of gene expression ratios observed by RNA-seq and qRT-PCR. The RNA-seq log2 (expression ratio) values (x-axis) are plotted against the log2 (expression ratio) obtained by qRT-PCR (y-axis). [file 12870_2015_435_MOESM6_ESM.tiff]

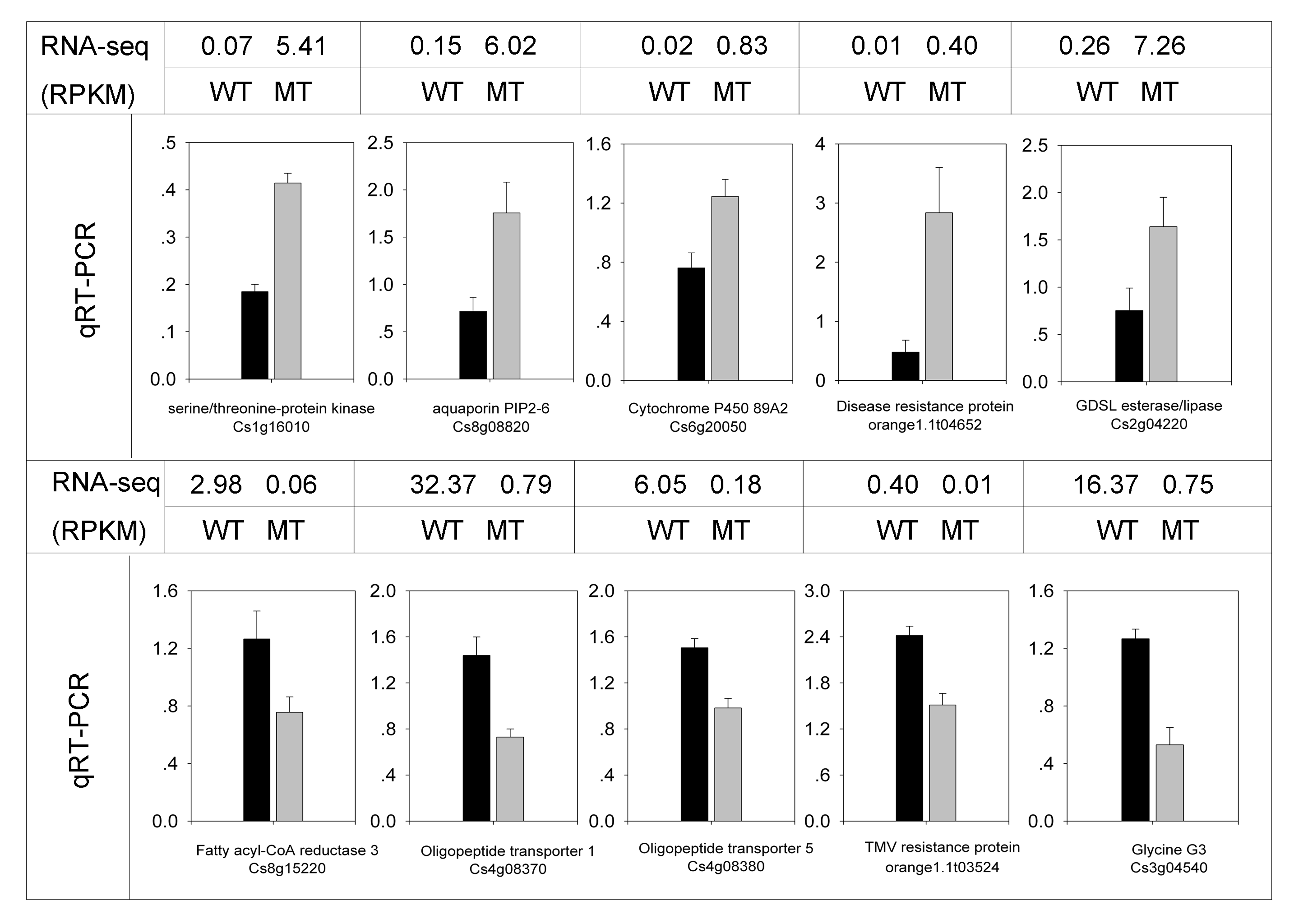

Supplement: Additional file 7: — Top 10 most DEGs in MT and WT. The transcript abundance from RNA-seq data was added on the top of each gene. RPKM, reads per kilo bases per million reads. The gene number refers to the sweet orange genome. [file 12870_2015_435_MOESM7_ESM.tiff]
