# Supplementary material for: Transcriptomic analysis of differentially expressed genes in an orange-pericarp mutant and wild type in pummelo (Citrus grandis)
Source: BMC Plant Biol. 2015 Feb 12;15:44. doi: 10.1186/s12870-015-0435-3 (PMC4352283; doi:10.1186/s12870-015-0435-3)
Supplement: Additional file 3: — Sequence information of carotenoid biosynthetic genes in MT and WT. [file 12870_2015_435_MOESM3_ESM.pdf]

GGPS

ATGAGTTGCGTCAATCTTGCGGCATGGACTCAAACATGCTCAATCTTTAACCAAGCTAGCAGTCGCAGA
TCTAACAAAACTCCGCCGTTCCGTACGCTTAAAACACTGCCCGTTTCCTTGGCTTCACAGAGACCAAGG
CGCCCCGTTTCGATCTCCGCAGTTCAAACTCTCGAGGAAAACCCCGCCCCTAGCCCCACTTTCGATTTC
AAGTCCTACATGATCCAAAAAGCAAGTACCGTCAACCAAGCCTTAGACGCCGCCGTTTCGCTCAAGGAC
CCCGTGAAGATCCACGAATCCATGCGATACTCTTTACTGGCGGGCGGCAAGCGGGTGCGCCCCGTGCTC
TGCCTCGCCGCGTGTGACCTTGTTGGTGGCCACGAGTCCATGGCCATGCCAGCTGCATGCTCTATCGAG
ATGATCCACACCATGTCCTTAATTCACGACGATTTGCCTTGCATGGATAACGACCCTCTTCGTCGAGGG
AAGCCCACGAACCACACGATCTACGGCGAAGACGTCGCCGTTTTGGCCGGCGATGCGCTTTTGGCCTAT
GCTTTTGAACATATCGCTGTTTGTACAAAAGGGGTTTCGCCTGCAAGAATTGTCAGAGCTATTGCGGAA
TTGGCTAAATATATCGGAGCTGATGGGCTTGTCGCTGGCCAAGTTGTTGATATCAATTCTGAAGGCCGA
AAAGGTTTGGGAATTGAGCATCTTGAATTTATACATGAACACAAAACTGCAGCTTTATTGGAGGCTGCT
GTTGTTATTGGAGCCATATTGGGCGGCGGAACTGACAACGAAGTCGAGAAACTGAGAACTTTTGCTCGC
TGTATTGGGCTTTTGTTTCAAGTAGTTGATGATATCCTCGATCTGACCAAGTCGTCAAAAGAATTGGGG
AAGACTGCTGGCAAAGATTTGGTGGCCGATAAGTTAACTTATCCCAAGTTGCTGGGGATCGAAGAATCG
AAGAAGTTAGCTGATAAGTTGAATAAAGATGCTCAACAGCAATTGTCGGAATTTGATCAGGAAAAGGCC
GCGCCTCTGATTGCTTTGGCTAACTATATTGCTTATAGGCAGAATTGA

PSY

ATGTCTGTTGCATTGCTATGGGTTGTATCACCTAACTCACAATTGTCCAATTGCTTCGGGTTCGTCGAT
TCAGTTCGAGAGGAAAACAGGCTGTTTTATTCATCAAGATTTCTTTACCAACATCAAACCCGGACTGCT
GTGTTTAATTCTAGACCTAAGCAGTTTAATAATAATAATAATAATAAGCAGAAACGGAATTCTTATCCT
TTAGATACAGATTTGAGGCATCCTTGCTCATCTGGAATCGACTTGCCTGAAATATCATGTATGGTTGCT
AGCACTGCTGGAGAAGTGGCCATGTCTTCAGAAGAAATGGTTTACAATGTTGTGCTCAAGCAGGCAGCC
TTGGTTAATAAGCAACCAAGTGGGGTTACTCGTGATCTTGATGTGAACCCAGATATTGCTTTACCCGGA
ACTTTAAGTCTGCTCAGTGAAGCTTATGATCGTTGTGGAGAAGTTTGCGCCGAGTATGCTAAGACATTT
TACTTGGGAACTTTGCTGATGACCCCTGAAAGGCGAAGGGCTATATGGGCTATATATGTGTGGTGTAGG
AGGACAGATGAGCTCGTTGATGGGCCTAATGCTTCACACATAACTCCAACAGCTTTAGACAGGTGGGAG
TCCAGGTTGGAAGACCTTTTCCGGGGTCGTCCATTTGATATGCTTGATGCTGGATTATCAGATACAGTA
ACCAAATTTCCTGTCGACATTCAGCCATTCAGAGATATGATAGAAGGAATGAGGATGGACCTTAGGAAG
TCAAGATACAAAAACTTTGATGAATTATACTTGTATTGTTATTATGTTGCTGGGACCGTAGGGCTAATG
AGTGTTCCAGTTATGGGCATAGCACCTGACTCACAGGCAACAACAGAGAGCGTCTACAATGCAGCATTG
GCACTAGGGATTGCTAATCAGCTCACTAACATACTCAGAGATGTTGGAGAGGATGCCCAAAGAGGAAGG
GTTTATCTACCACAAGATGAGTTGGCACAGGCGGGGCTTTCAGATGATGACATATTTGCTGGAGAAGTG
ACTAATAAATGGAGAAACTTCATGAAGAACCAAATTAAGAGGGCAAGGATGTTCTTTGATATGGCTGAG
AACGGTGTGACCGAGCTGAGTGAAGCTAGTAGATGGCCGGTATGGGCTTCATTGCTGTTGTACCGGCAA
ATACTGGATGAGATTGAGGCCAATGATTACAACAACTTCACAAAGAGAGCTTATGTGAGTAAAGCCAAG
AAGATAGCTGCACTACCAATTGCATATGCAAAATCCCTCTTACGCCCGTCAAGAATATATACCAGTAAG
GCTTAA

PDS

ATGAGCCTTTGCTTCAGCGTTTCTGAAAGTGCTTTCAACTTGCGATATGGTTTCCGAGATAGTGAACCG
ATGGGTCAGAGCCTGAAAATTCGAGTTAAAACGAGGACAAGGAAGGGTTTCTGTCCTTCGAAGGTGGTT

TGTGTGGACTACCCAAGACCAGATATTGATAATACATCTAATTTCTTGGAAGCTGCTTACTTATCTTCG
TCATTTCGTACTTCTCCTCGTCCTTCTAAGCCGTTGAAAGTTGTAATTGCTGGTGCAGGTTTGGCTGGT
TTATCAACTGCAAAATATTTGGCAGATGCAGGCCACAAGCCTTTGTTACTGGAAGCAAGAGATGTTCTA
GGTGGAAAGATAGCTGCCTGGAAAGATGGGGACGGGGACTGGTATGAGACAGGCCTTCATATTTTCTTC
GGGGCTTACCCAAATATACAGAACCTGTTTGGAGAACTTGGTATTAATGACCGGTTGCAGTGGAAGGAG
CACTCTATGATTTTTGTAATGCCAAACAAGCCCGGAGAATTCAGCCGATTTGATTTTCCTGAAGTTCTT
CCAGCTCCGCTAAATGGGATATTGGCCATTTTAAGGAACAATGAAATGCTGACTTGGCCGGAGAAAGTG
AAGTTTGCAATTGGACTGCTTCCAGCAATAATTGGTGGACAGGCATATGTTGAAGCTCAAGATGGTTTA
ACTGTTCAGGAGTGGATGAGAAAGCAGGGTGTACCTGATCGAGTGACGACGGAGGTGTTTATTGCCATG
TCAAAGGCACTAAACTTCATAAACCCTGATGAACTGTCAATGCAATGTATATTGATTGCCTTAAACCGA
TTTCTTCAGGAGAAGCATGGTTCGAAGATGGCATTCTTAGATGGCAACCCCCCAGAGAGACTTTGCTTG
CCTATTGTTGAACACATTCAGTCACTGGGTGGTGAAGTCCGGCTTAATTCCCGAGTTCAGAAAATTGAG
CTCAATGATGATGGAACTGTGAAGAATTTTTTACTAACTAATGGCAATGTGATTGACGGAGATGCTTAT
GTATTTGCCACACCTGTTGATATCCTCAAGCTTCAGTTACCTGAAAACTGGAAAGAGATGGTATACTTC
AAGAGATTAGAGAAATTGGTGGGAGTTCCAGTCATCAACATCCACATATGGTTTGACAGGAAATTGAAA
AACACTTATGATCACCTACTCTTTAGCAGAAGTCCCCTTCTAAGTGTGTATGCCGACATGTCTTTAACT
TGTAAGGAGTATTACAACCCCAATCAATCCATGCTGGAGTTAGTTTTTGCCCCGGCTGAAGAGTGGATC
TCATGCAGTGACTCAGAAATCATTGATGCTACAATGAAGGAGCTTGCAAAACTATTTCCTGATGAAATT
TCTGCTGATCAGAGCAAAGCAAAGATTGTGAAGTACCATGTCGTCAAAACGCCAAGGTCTGTATATAAA
ACCATCCCAAATTGTGAACCTTGCCGTCCCTTACAAAGGTCTCCTGTAGAAGGGTTTTATTTAGCCGGG
GATTACACAAAACAGAAGTATTTGGCTTCAATGGAAGGTGCTGTTTTGTCAGGGAAGCTTTGTGCACAA
GCAATTGTACAGGACTATGTGCTGCTTGCTGCACGGGGGAAAGGGAGATTGGCTGAGGCAAGCATGTGT
TAA

CRTISO

ATGTTCATCTCTTGTTGCTCTCTTTCCATACCTGAATTAAATTTCACCCCTTCACTTATAAACCATAAT
TCTCAGGTTTTGATTCACCAAAATAGGTCGAAGTTTACTGCTTTAAGTAATAAAGTTCAGTCCTTTGGT
CAGCGTGACGCTATGCAGTTGGGTAGTAGCAGCAAACCCAGAATCCGTAATTGTATGATTCAATCTTTT
GAACATGTAAATACTCTACCTTTCAGTGATTACAAGTTCAGTAGGATGAAACTATTCAAACCCAGATAT
GAAAAAAGCAGTCTTTTCTCTGGGGATTCACTAAAAAGCTCAAATTTCAATGGTTCCACCCTGAGGAGT
GAGGATTTGGGTTGTGGGGAAAGTGAGAGGAATAGGGACTTTGCTTTGATGGCAAAGACAGTGATGAGT
GTTGATAATTTGGTGGAGATAGGGGGAAATGAAGGAATGAGTAGAGGAGCTGATGACTATGATGCAATT
GTTATAGGGTCAGGTATTGGGGGCTTGGTAGCAGCTACACAGCTGGCAGTGAAGGGAGCTAGGGTTTTG
GTGTTGGAGAAGTATGTGATTCCTGGTGGAAGCTCCGGGTATTATGAGAGGGATGGGTATACTTTTGAT
GTTGGTTCTTCTGTGATGTTTGGGTTCAGCGATAAGGGCAATCTAAATTTGATAACCCAAGCACTGGCA
GCTGTTGGTTGTGAGATGGAGGTGATACCTGATCGAACCACTGTCCATTTCCACCTACCAAATGACCTT
TCTGTTCGAGTTCATAGAGAATATAGTGATTTTGTTGCAGAGCTTACCAGTAAATTTCCTCATGAAAAG
GAAGGAGTCCTTGCATTTTACGGTGAATGTTGGAAGATCTTCAATGCCTTGAACTCATTGGAGCTGAAG
TCTCTTGAAGAGCCAATCTACCTTTTTGGACAGTTCTTTAAGAGGCCTCTTGAATGCTTGACACTTGCC
TATTATCTGCCTCAGAATGCTGGAAACATAGCTCGAAAGTACATAAAGGATCCACAGCTGTTGTCCTTC
ATAGATGCAGAGTGCTTTATAGTGAGCACAATCAATGCTTTGCAGACACCAATGATCAATGCTAGCATG
GTTTTATGTGACAGGCATTTTGGAGGGATTAACTACCCTGTTGGTGGTGTCGGTGGAATCGCAAAGTCA
TTAGCAAAAGGCCTGGCTGATAAGGGCAGTGAAATACTCTATAAGGCTAATGTAACCAAAGTTATACTT
GAGCAAGGCGAAGCTGTAGGAGTGAGACTATCAGATGGAAGGGAGTTTTATGCCAAAACCATAATATCC

AATGCTACTAGATGGGATACATTTGGGAAGCTATTAAAAGGAGAACAACTTCCAAAAGAAGAAGAAAAT
TTTCAGAAACTATATGTCAAGGCTCCTTCGTTTCTTTCCATTCACATGGGTGTTAAAGCTGAGGTTCTG
CCACCTGACACAGATTGCCACCATTTTGTGCTTGAGGATGACTGGAACAGACTGGAGGAGCCTTATGGA
AGTATATTTTTAAGCATTCCAACGGTTCTTGATTCATCTTTGGCTCCAGAGGGGCACCATATTCTTCAC
ATATTTACAATTTGTTCCATAGAGGACTGGGAGGGACTGGCTCAAAAGGACTATGATGCAAAGAAGGAG
CTTGTGGCAGATGGAATAATCAATAGACTGGAGAACAAACTGTTTCCAGGGCTTAAACAATCAATTGCT
TTTAGGGAGATTGGGTCACCAAAGACACACCGGCGGTATCTAGCTCGCGATCAGGGTACCTATGGGCCA
ATGCCTCGTGGAACACCCAAGGGCTTGTTGGGGATGCCATTTAACACAACAGGGATAAATGGTCTTTAC
TGTGTTGGAGATAGCTGCTTTCCAGGACAAGGTGTAATAGCTGTTGCATTTTCTGGAGTAATGTGCGCT
CATCGAGTAGCTGCTGATATTGGGCTTGAGAAGAAGTCCCCAGTACTGGATGCTGGTCTCCTTCGATTA
CTTGCTTGGTTAAGGAGTTTGGCATGA

LCYB

ATGGATACTTTACTCAAAACTCATAACAAGCTTGAATTCTTGCCCCAAGTTCACGGGGCTTTGGAAAAA
TCCAGTAGTTTAAGCTCATTGAAGATTCAGAACCAGGAGCTTAGGTTTGGTCTCAAGAAGTCTCGTCAA
AAGAGGAATAGGAGTTGTTTCATTAAGGCTAGTAGTAGTGCTCTTTTGGAGCTAGTTCCTGAAACCAAG
AAGGAAAATCTTGAATTTGAGCTTCCCATGTATGACCCATCAAAGGGCCTTGTTGTAGACCTAGCAGTT
GTCGGTGGCGGCCCGGCTGGGCTTGCTGTTGCTCAGCAAGTTTCAGGGGCGGGGCTTTCGGTTTGCTCG
ATTGATCCATCTCCCAAATTGATTTGGCCAAATAATTATGGTGTTTGGGTGGATGAATTTGAGGCCATG
GATTTGCTTGATTGCCTTGATACTACTTGGTCTGGTGCTGTTGTGCACATTGATGATAATACAAAGAAG
GATCTTAATAGACCTTATGGGAGAGTTAATAGGAAGTTGCTGAAGTCGAAAATGCTGCAAAAATGCATA
ACCAATGGTGTTAAGTTTCACCAAGCTAAAGTTATTAAGGTTATTCATGAAGAGTCCAAATCTTTGTTG
ATTTGCAATGATGGTGTGACAATTCAGGCAGCCGTGGTTCTTGATGCTACGGGGTTCTCTAGGTGTCTT
GTCCAGTATGATAAGCCCTATAATCCAGGTTACCAAGTGGCATATGGAATACTAGCTGAGGTAGAACAG
CACCCGTTTGATTTAGACAAGATGGTTTTCATGGATTGGAGAGATTCGCATCTGAACAACAATTCGCAG
CTCAAAGAGGCAAATAGCAAAATTCCTACTTTTCTTTATGCCATGCCCTTTTCGTCAAACAGGATATTT
CTTGAAGAGACTTCGCTAGTGGCGCGGCCTGGAGTGCCAATGAAAGATATCCAGGAAAGAATGGTGGCT
AGATTAAAGCACTTAGGCATAAAAGTTAAAAGCATTGAAGAGGATGAGCATTGTGTCATTCCGATGGGT
GGGCCCCTTCCAGTGCTTCCTCAAAGAGTTGTTGGAATAGGTGGTACCGCTGGGATGGTGCACCCTTCA
ACTGGCTATATGGTGGCAAGGACTTTAGCTGCGGCTCCTATTGTTGCAAATGCAATCGTTCGAAGCCTC
AGTTCTGACCGAAGCATTTCAGGACACAAATTGTCTGCTGAAGTTTGGAAAGATTTGTGGCCCATAGAA
AGGAGAAGGCAAAGGGAGTTCTTCTGTTTTGGTATGGATATCCTGCTCAAACTTGACTTACCTGCCACT
AGAAGGTTTTTCGATGCTTTTTTTGATCTGGAGCCTCGTTATTGGCATGGTTTCTTATCATCGAGATTG
TTTCTCCCCGAGCTTTTAGTTTTTGGGCTTTCTCTATTCTCACATGCCTCTAATACTTCTAGGCTAGAG
ATCATGGCAAAGGGCACTCTTCCTTTGGTTAACATGATCAACAACTTGGTACAAGATACAGATTAA

LCYE

ATGGAATACTACTGTCTTGGAGCTCGAAATTTCGCAGCAATGGCGGTATCTCCTTTCCCAACAGGGCGG
ACGCAAAGGAAAGCACTGAGAGTGAGAACCAAACAGAGTGCTGTAGATTGTAATCATAGTTCCTATAAA
GTGACAGCTAGAGCTACTAGTAGTAATGCTGGTAGTGAGAGTTGCGTAGCCGTCAAAGAAGAAGATTAT
ATTAAGGCCGGTGGCTCTCAGCTTGTTTTCGTACAAATGCAGCAAAACAAGTCAATGGATAAACAGTCT
AAATTAGCCGATAAGCTACCGCCAATATCAATTGGTAATGGTATTTTGGATTTGGTGGTGATTGGTTGT
GGCCCAGCTGGTCTTGCTTTGGCTGCAGAATCAGCGAAGTTGGGATTAAATGTTGGACTTATTGGCCCG
GATCTCCCTTTCACAAACAATTATGGTGTGTGGGAAGATGAATTTAGAGATCTTGGACTTGAAGGGTGT

ATCGAACATGTCTGGAGAGACACAGTTGTATATATTGATGAAGATGAACCCATCTTGATTGGTCGTGCT
TATGGACGAGTTAGTCGACATTTGCTTCATGAAGAATTATTAAGAAGGTGTGTCGAGTCAGGTGTTTCA
TATCTTAGCTCAAAAGTGGAAAGCATTACGGAATCTACCAGTGGTCATCGTCTTGTAGCTTGTGAACAT
GATATGATTGTCCCCTGCAGGCTTGCTACTGTTGCTTCTGGAGCAGCATCAGGGAAGCTATTGGAATAT
GAGGTGGGGGGTCCCAAAGTTTCTGTCCAAACAGCTTATGGTGTGGAGGTTGAGGTGGAAAATAATCCA
TATGATCCAAGCCTTATGGTTTTCATGGACTACAGAGACTGTACTAAGCAAGAAGTTCCATCTTTTGAA
TCTGACAATCCAACATTTCTTTATGTCATGCCCATGTCTTCAACAAGAGTTTTCTTTGAGGAAACTTGT
TTGGCATCGAAAGATGGTTTACCTTTTGACATATTGAAGAAAAAGCTCATGGCAAGGTTAGAGAGATTG
GGAATCCAGGTTTTGAAAACTTATGAAGAGGAATGGTCATATATTCCAGTTGGTGGTTCCTTACCGAAT
ACAGAACAAAGAAACCTCGCATTTGGTGCTGCTGCTAGCATGGTGCATCCAGCCACTGGCTACTCAGTA
GTCAGATCACTGTCAGAAGCTCCAAACTATGCTTCTGCAATTGCATATATATTGAAACACGATCATTCC
AGAGGTAGACTTACACATGAACAAAGTAATGAGAATATCTCAATGCAAGCTTGGAATACTCTCTGGCCA
CAGGAAAGGAAGCGCCAAAGAGCTTTTTTTCTCTTTGGACTAGCACTCATTTTGCAACTGGATATTGAG
GGCATCAGGACATTCTTTCGCACTTTCTTCCGATTACCCAAGTGGATGTGGCACGGTTTCCTTGGTTCT
AGTCTCTCATCAGCCGATCTCATTCTATTTGCCTTCTATATGTTTATTATAGCACCAAATGATCTGAGA
AAGTGCCTTATCAGACATCTAGTTTCAGATCCAACTGGAGCAACTATGGTAAGAACATACCTGACTTTA
TAG

LCY2B

ATGGCAACTCTTCTTAGCCCGTTTTCTCCTTCTCCTTTAGCTAAAGTTTCGCAAATAATTGATTCAACA
TCATCACCTTCATTTTCCCTATTTCCATTAGGCCGCCAAAATGCATGTTCGAGAAAGGCGGATCATCAT
CATCATCACAGGATCCGGACAAGCAAGTTTGGTAACTTCCTAGAGTTGACACCGGAGTCGGTACCTGAA
TTCTTAGACTTTGATCTCCCCTGGTTTCATCCGTCCGATCGTATTCGATATGACGTGATCATCATTGGC
ACTGGACCAGCCGGCCTCCGTCTAGCTGAGCAAGTCTCATCGCGTCATAGTGTCAAGGTATGTTGTGTT
GATCCTTCACCTCTTTCTACGTGGCCTAACAACTATGGAGTTTGGGTTGATGAGTTTGAAGACATAGGA
CTTGTAGACTGTTTGGACAAAACTTGGCCGATGACTTGTGTTTTTATTAATGATCACAAGACCAAGTAT
CTAGACAGGCCCTACGGTCGTGTTAGTAGAAATATTTTGAAGACAAAGTTATTAGAGAATTGTGTTTCA
AATGGAGTTAAGTTTCATAAGGCTAAAGTTTGGCATGTGAATCATCAGGAGTTCGAGTCTTCGATTGTT
TGTGATGATGGGAATGAGATTAAGGCTAGCTTGATTGTTGATGCTAGTGGCTTTGCTAGTAGTTTTGTT
GAGTATGATAAGCCAAGAAACCATGGATACCAAATTGCTCATGGGATTTTAGCTGAGGTTGAGAGTCAC
CCTTTTGATTTAGACAAAATGGTTCTCATGGATTGGAGAGATTCCCATTTAGGGAATGAGCCTTACTTG
CGAGCTAGCAATTTGAAGCTCCCAACTTTTCTCTATGCAATGCCATTTGATTCAAATTTGGTATTTTTA
GAAGAAACATCTTTGGTTAGTAGGCCAGTTTTGTCATATAAAGAGGTTAAGAGCAGAATGGCAGCGAGG
TCAAGGCATATGGGAATTAGAGTTAAAAGAGTGATTGAAGATGAAAAATGTTTGATTCCAATGGGAGGT
CCTCTGCCTGTGATCCCACAAAGTGTGATGGCTATTGGCGGCACGTCTGGTTTAATCCATCCTGCAACT
GGGTATATGGTGGCTCGGACCATGGCTCTGGCCCCTGCCTTGGCTGATGCAATAGCTGAATGCCTTGGC
TCAACCAGGATGATCAGAGGCAGGCCACTTCATCAGAAAGTGTGGAATGGGTTGTGGCCAATTGACAGA
AGATGCAATAGGGAGTTTTATTCATTTGGTATGGAGACTTTGTTGAAGCTGGATTTGAAGGGGACTAGG
AGATTCTTTGATGCTTTCTTTGATTTGAATCCTTACTACTGGCATGGGTTTCTGTCCTCAAGGTTGTCT
CTTGCAGAGCTTGCTGGCCTAAGCTTGTCTCTCTTTGGACACGCCTCGAATTCTTCCAGGTTGGATATT
GTTACCAAGTGCCCTGTTCCTCTGGTTAAAATGATGGGGAATCTTGCCCTTGAAACCATTTGA

BCH

ATGGCGGTCGGACTATTGGCCGCCATAGTCCCGAAGCCCTTCTGTCTCCTCACAACAAAACTTCAACCC

TCTTCGCTCCTCACAACAAAACCCGCTCCCCTTTATGCCCCTCTCGGTACCCACCACGGCTTCTTTAAT
GGCAAAAACCGAAGAAAACTCAACTCTTTCACCGTATGTTTTGTTTTAGAGGAGAAAAAACAAAGCACC
CAGATCGAGACTTTCACGGAGGAGGAGGAGGAGGAGTCGGGTACCCAGATCTCGACTGCTGCCCGCGTG
GCCGAGAAATTGGCGAGAAAGAGATCCGAGAGGTTCACTTATCTCGTTGCTGCCGTCATGTCTAGTTTT
GGTATCACTTCCATGGCTGTCATGGCTGTTTATTACAGGTTCTGGTGGCAAATGGAGGGTGGAGAGGTG
CCTTTAGCTGAAATGTTTGGCACATTTGCTCTCTCTGTTGGTGCTGCTGTGGGCATGGAGTTTTGGGCA
CGATGGGCTCATAAAGCTCTGTGGCATGCTTCTTTATGGCATATGCACGAGTCTCACCATCGACCAAGA
GAGGGTCCTTTTGAGCTAAACGATGTGTTTGCCATAATCAACGCAGTTCCAGCCATAGCCCTTCTCTCT
GTTGGCTTCTTCCACAAAGGCCTTGTACCTGGTCTCTGTTTTGGTGCTGGACTTGGCATTACGGTGTTT
GGGATGGCCTACATGTTCGTCCACGATGGTCTCGTTCACAAAAGGTTCCCTGTGGGTCCCATTGCCGGC
GTGCCTTATTTCCGGAGAGTCGCTGCTGCTCACCAGCTTCACCACTCGGATAA

CCD4c

ATGGATGCCTTGTCTTCCTCCTTCATGTCAACATTACCACCTCAAATACAGCGGCTCCACTACTCTCCA
ACCAAAACAAAAGCACCACTGTTGTTACCATCATCATCATCTTCATCGCGAACCAATAATGTGATAGTT
TCAACAACATCACCACCCAAAAAACCTGAAACCACAGCAGAAAATAACAAACAGCAAACAAAGACGACG
CAGAAACCATCATCATCAGTATCAACGACAACAAACGTAAGTGCAGTGATACTTAATACACTCAACAAA
TTGATAAAACCTAAACTCCAGGCGCAACCACTGTCTCTCGACCCAAGTCGTGCATTTTCCAATAACTTA
GCTCCTGTCGATGAGCTTCATCCAACACAATGCCAGGTAACACAAGGGTCTCTCCCGTCCTGCTTAGAG
GGTGCATACATTCGTAATGGCCCTAACCCTCAATACCCTCCTCGTTCAGAGCCTTATAGTCCCTTCGAC
GGCGATGGCATGCTGCACTGCATTAAAATCTCACAAGGCCAGGCCACCTTCTGTAGCCGCTATGTCAAA
ACTTATAAATACACCATTGAAAACACAGTTGGTTCTCCCATTGTTCCGCGTCCCTTCTCTAGCTTCAGT
GGCCCTCTTGCGTTTTTAACAAGAGCTGGTGTCTTAGCAGCTCGTGTGCTCACAGGTCAACTTAGTATT
AACAGCGGATTTGGTCTAGCGAACACCAGTTTAGCTTTCTTGGGAAACCGTTTGCTTGCACTTTATGAA
GGTGATCTCCCCTATGCTGTGCGCTTGACGTCTAATGGTGATATTGAAACTTTGGGTCGTTGTGATTTG
GATGGAAAGTTAGTGATGAACATGACTGCCCATCCCAAGAAAGACCCCGAAACAGGCGAAATGTTTGCT
TTCCGTTATAGTCCAATGCCTCCGTTTGTGACCTACTTCTGGTTTGATGCTAACGGAGAGAAACAACCT
GACGTGCACATAAAAATGCCAGGTCAATGGCTTCGTCACCCTCCTTTAATGCATGACTTTGCAATAACA
AAGAGATACGCTATATTTGTCGACACACAAGTGCAGGCTTATGGTATGGAAATGATTCTTCGACGGGAG
CCTCTGCTCCATTCGGATCCAACAAAGGTGTCAAGGATTGGTGTTATGCCTCGATATGCAAAAAATTCA
TCGCAAATGAGATGGTTTGATGCGCCGGGACTTAACAATATACATGCCGTCAATGCATGGGATGAGGAG
GATGGCAATGTAATAGTCATGGTGGCACCAAATATATTATCTGTGGAACAAGTGTTTGAAAGAATGGAA
CTTATTCATTGTTTGATGGAAAAAGTGAGGATTGATCTCAGAACAGGAGTAGTTTCAAGGCATCCTATT
TCAGCAAGAAATTTGGATATGGCGGTTATAAATCCGGGATATGTGGCAAAAAAGAACAGGTATGTGTAT
GCAGCAGTGGGCGATCCAATGCCTAAGATGACAGGCTTGGTGAAGCTGGATTTGAAGAAAGGGGAACGA
CGGGAGTGCACTGTGGCTACCAGGATTTACGGGCCGGATTGCTACGGCGGCGAGCCGTTCTTCGTGGCC
AGAGACGCAGCGGACAAAGAAGCAGATGAAGATGATGGCTACATTTTGTCGTATGTTCATGATGAGAAG
GCAGGAGAATCAAAGTTCTTGGTGATGGATGCAAAATCACCTCACTTGGACATTGTGGCTGAAGTGAAG
CTGCCTCAGCGGGTGCCTGCAGGCTTTCATGGCCTTTTTGTGAGGGAAATCGACTTCTGTAAGCTTTGA

NCED2

ATGGCTGCTTCTACTACATCACCCAACTCTTGTAGTTGGGGTAAATCTCAAATTCTTAACCCATTCTCT
TCATCTTTGGTTGACAAGGGCTTCCCAACGAAGTCCATAGCCTTGAAGAAGAAGCCCAACAAATCCTTG
AAAGTAGAATCTGCCTTACACTCTCCTTCAGTCCTTCATTTCCCTAAACAGCCTTACCCGAATCCTGTA

ATTACCAGACCGGACTCTACAGCAAAACCTGACAACAAGAGCAACAAAAGCCAAGGACAAACACAGCAA
TCACAATGGAATCCCCTCCAAAGAGCCGCTGCCATGGCCTTGGACATGGCGGAGAACGCGTTGCTCTCA
CGAGAGCGCCAACACCCACTTCCCAAAACAGCCGACCCACGTGTCCAAATTGGCGGTAACTTCGCTCCG
GTGCCGGAACGGCCCGTCCGGCATGGTCTGCCGGTCACTGGCTCCATCCCCGAATGTATCAACGGCGTT
TACCTCCGCAACGGTGCCAACCCATTATTCGAGCCGGTTGCCGGTCACCATTTCTTTGACGGTGACGGT
ATGGTCCATGCAGTCACCATCGGTAACGGAAGCGCAAGTTACGCTTGTCGTTTCACAGAGACACAGAGA
TTAATGCAGGAGAGAGAATTAGGGCGTCCTGTTTTTCCAAAAGCAATAGGCGAATTACACGGACACAAT
GGTATTGGGCGTCTCTTGCTTTTCTACGCAAGAGGATTGTTCGGACTCGTTGATCATAAACAAGGCACT
GGTGTGGCAAACGCTGGCTTAGTTTACTTCAATAACAGGCTTTTAGCAATGTCCGAAGATGATCTTCCA
TACCAAGTACGCATCACTCCTTCTGGGGATCTTGAAACCGTTGGGCGTTACAATTTTAACCACCAACTT
AAGTCAACAATGATAGCTCATCCGAAAGTTGATCCCGTATCAAAGGAGCTTTTTGCTTTGAGTTATGAT
GTTGTTCAAAAGCCTTATTTAAAATACTTTAGATTTTCTGCTGATGGAGACAAATCACCCGACGTAGAG
ATTCATCTGCCAGTGCCCACCATGATGCACGATTTCGCTATCACTGAGAATTTTGTGGTGATCTCGGAC
CAACAAGTCGTTTTCAAGCTCCAAGAAATGATAACGGGTGGCTCTCCGGTGATTTATGACAAGAACAAG
AAGTCCCGGTTCGGGATTCTTGCAAAGAATGCTAAAGATTCTAACGACATCATCTGGATTGAATCACCG
GACACGTTCTGCTTTCACTTGTGGAACGCTTGGGAGGAGCCGGAAACTGATGAAATTGTTGTCATTGGA
TCATGCATGACACCTGCTGACTCAATTTTCAACGAGTGTGACGAGAGTCTGAAGAGTGTTTTATCCGAA
ATTCGGCTCAATTTAAAGACTGGTGAGTCCACGCGCCGCCAGATTCTCTCGGAGTCTGATCAAGTGAAC
TTGGAGGCTGGGATGGTGAATCGCAACAGCCTTGGTAGAAAAACTCAGTTCGCTTATCTAGCCATTGCG
GAGCCATGGCCTAAAGTCTCAGGTTTTGCTAAAGTGGATCTCTCTTCAGGAGAGGTTAAAAAGTATTTT
TACGGCGATAATAAATATGGCGGCGAGCCATTTTTCTTGCCAAGAGATGCTAATAATTCTGAAAATGAA
GACGATGGTTATATTCTTGCATTTGTTCATGACGAGAAGACTTGGAAATCAGAGCTGCAAATTGTGAAT
GCCATGAATTTACAGCTTGAAGCTTCGATTAAGTTACCTTCGAGAGTACCATACGGTTTTCATGGAACT
TTTGTTGACTCCAAGGACTTGGTGAATCAAGCATAA

NCED3

ATGGCGGCAGCAACTACTACTTCTTCACTCATAGACTTGGGCTCTTGTAAAGATTCTTGTTCTTCTCCT
TCTTTTTCTTCTTCAAGAGCAAGAATTGCTTTTAGTTTAAAAAAGCCCACCGAAATCACTTGCTCTCTT
CAAACACCTTCCATTCTTCATTTCCCCAAGCAGTCCCCAAAATACCCTCCATCACCTGCAGCTACCCCT
CCTCCATCTTCTCCTCCTCTTACAACTAAGCAGCAACCCAAGGAAAATGACAATTTCGCCCCTTCAAAA
TGGAACTTCTTACAAAAAGCAGCAGCTTTAGCCTTGGACGCAGTCGAAAACGCATTGGTTTCTCAAGAG
CGTCAGCATCCTCTCCCCAAAACAGCCGACCCCACCGTCCAAATCGCCGGAAACTTCTCTCCGGTGCCC
GAAAAACCCGTCTGCCAGAACCTTCCCACTACCGGAAAGGTCCCCGACTGCATTCAAGGCGTCTACGTC
AGAAACGGAGCCAACCCACTTCACGAACCGGTCGCCGGCCACCATTTCTTCGACGGAGACGGCATGGTT
CATGCAGTCAAATTCAACAAAGGCTCTGTCAGCTATTCTTGTCGTTTCACTGAAACAAACCGTTTTGTT
CAAGAACGTAGCTTAGGCCGCCCCGTATTCCCCAAAGCCATTGGCGAGCTTCACGGCCACACGGGCATC
GCAAGATTGCTTCTATTCTACAGCAGAGCGCTCTTCGGTCTCGTTGACCCCAGCCACGGCACTGGCGTT
GCCAACGCCGGCCTTGTTTACTTCAACAACCGTTTGTTGGCCATGTCTGAAGATGACTTGCCTTATCAG
GTGCGCGTCACTCCATCCGGCGAGCTCAAAACAGTCGGCCGTTTCGACTTCAGCGGCCAGCTCAAGTCC
ACGATGATAGCTCATCCGAAAGTTGATCCCGTGACGGGTGATTTGTTTGCTCTGAGTTATGACGTTGTC
AAGAAGCCTTACTTGAAGTACTTTCAGTTTTCGCCCCAAGGGATCAAGTCTCCGGACGTTGAGATTCCC
CTTGAGGAGCCTACAATGATGCATGATTTCGCAATCACTGAGAATTTTGTTGTGGTGCCTGACCAGCAA
GTGGTGTTCAAGTTGAAAGAGATGATCCGAGGTGGCTCCCCTGTGATTTATGACAAGAACAAGGTGTCA
AGATTTGGGATTTTGGACAAGAATGCCACCGATGCTTCCAAAATGAAGTGGATTGACGCCCCTGATTGC

TTCTGTTTCCATCTTTGGAATGCTTGGGAAGAGCCTGAGAGGGATGAAGTTGTTGTAATTGGGTCCTGT
ATGACTCCTGCCGACTCCATTTTCAACGAATGTGATGAGAGTCTGAAGAGTGTTTTGTCCGAAATTAGA
CTCAATCTAAAGACCGGTGAGTCCACTCGCCGCCCCATAATCTCCGAGGATGAGCAAGTGAACTTGGAA
GCCGGAATGGTGAACAGAAACTTGCTCGGAAGAAAGACCCGTTTCGCGTACTTAGCCCTCGCCGAGCCA
TGGCCTAAAGTTTCAGGTTTTGCCAAAGTTGATCTCTTAACCGGACAAGTAAACAAGTTTATTTACGGA
GATCAAAGGTACGGGGGCGAGCCTTTGTTCTTTCCGAGAGACCCCAATTCGGAGAATGAGGATGATGGC
TACATTCTCGCTTTTGTACACGATGAAAAGGAGTGGAAATCAGAGCTGCAAATTGTTAACGCAATGACT
CTGGAGCTTGAAGCTACAGTTAAGCTTCCATCTCGAGTTCCTTATGGCTTCCACGGGACGTTCATAGGC
GCGAAAGATTTGGCCAAGCAGGCCTAA
